# Insights into the Genome Sequence of *Chromobacterium amazonense* Isolated from a Tropical Freshwater Lake

**DOI:** 10.1155/2018/1062716

**Published:** 2018-05-20

**Authors:** Alexandre Bueno Santos, Patrícia Silva Costa, Anderson Oliveira do Carmo, Gabriel da Rocha Fernandes, Larissa Lopes Silva Scholte, Jeronimo Ruiz, Evanguedes Kalapothakis, Edmar Chartone-Souza, Andréa Maria Amaral Nascimento

**Affiliations:** ^1^Departamento de Biologia Geral, Instituto de Ciências Biológicas, Universidade Federal de Minas Gerais, Belo Horizonte, MG, Brazil; ^2^Centro de Pesquisas René Rachou, FIOCRUZ, Belo Horizonte, MG, Brazil

## Abstract

Members of the genus *Chromobacterium* have been isolated from geographically diverse ecosystems and exhibit considerable metabolic flexibility, as well as biotechnological and pathogenic properties in some species. This study reports the draft assembly and detailed sequence analysis of *Chromobacterium amazonense* strain 56AF. The de novo-assembled genome is 4,556,707 bp in size and contains 4294 protein-coding and 95 RNA genes, including 88 tRNA, six rRNA, and one tmRNA operon. A repertoire of genes implicated in virulence, for example, hemolysin, hemolytic enterotoxins, colicin V, lytic proteins, and Nudix hydrolases, is present. The genome also contains a collection of genes of biotechnological interest, including esterases, lipase, auxins, chitinases, phytoene synthase and phytoene desaturase, polyhydroxyalkanoates, violacein, plastocyanin/azurin, and detoxifying compounds. Importantly, unlike other *Chromobacterium* species, the 56AF genome contains genes for pore-forming toxin alpha-hemolysin, a type IV secretion system, among others. The analysis of the *C. amazonense* strain 56AF genome reveals the versatility, adaptability, and biotechnological potential of this bacterium. This study provides molecular information that may pave the way for further comparative genomics and functional studies involving *Chromobacterium*-related isolates and improves our understanding of the global genomic diversity of *Chromobacterium* species.

## 1. Introduction

The genus *Chromobacterium*, belonging to the class Betaproteobacteria and family Chromobacteriaceae (formerly Neisseriaceae) [1], was originally proposed in 1881 by Bergonzini [2]. Until 2007, the only validated species within the genus *Chromobacterium* was *C. violaceum*, the type species of the genus. The current taxonomy of *Chromobacterium* consists of 11 recognized species: *C. violaceum* [2], *C. subtsugae* [3], *C. aquaticum* [4], *C. haemolyticum* [5], *C. piscinae*, *C. pseudoviolaceum* [6], *C. vaccinii* [7], *C. amazonense* [8], *C. rhizoryzae* [9], *C. alkanivorans* [10], and *C. sphagni* [11]. It is evident that, since 2007, there has been a rapid taxonomic expansion of the *Chromobacterium* genus.

Species in this genus have been collected from geographically diverse ecosystems. The wide spread of *Chromobacterium* throughout a variety of environments, such as soil, water, and plant from tropical and subtropical regions, is due to its considerable metabolic flexibility [12–16]. *Chromobacterium* has attracted considerable biotechnological interest for its potential as a biocontrol agent, environmental detoxification, and bioprospecting, in addition to industrial and pharmacological uses. Among its important characteristics, there are the production of chitinase (with fungicide, insecticide, and nematicide activities), polyhydroxyalkanoates (biodegradable plastics), violacein (with anticarcinogenic and antimicrobial activities), and cyanide biogenesis associated with gold recovery production [17]. These attributes were confirmed by the complete sequencing of the first strain of the type species *C. violaceum*, ATCC 12472, and by further genomic studies of *C. vaccinii*, *C. subtsugae*, *C. haemolyticum*, *C. piscinae*, *C. aquaticum*, and *C. pseudoviolaceum*, which showed alternative pathways for energy generation, a large number of open reading frames (ORFs) for transport-related proteins, and systems for stress adaptation, indicating the possible versatility and adaptability of this bacterium. Moreover, in spite of the fact that *Chromobacterium* is mainly considered a free-living microorganism, it is recognized as an important opportunistic pathogen and occasionally leads to lethal infections in mammals [18, 19].

Despite the potential importance of *Chromobacterium* in industry, agriculture, and medicine, few genomic analyses have been performed to gain insights into the biotechnological applications and pathogenicity of *Chromobacterium* species. Here, we present high-quality draft genome sequence of *C. amazonense* strain 56AF, isolated from a tropical freshwater lake. This organism was selected for sequencing due to its high resistance to *β*-lactam and as part of an ongoing effort to investigate the bacterial diversity on protected area (undisturbed environment). Additionally, a comparative genomic analysis of *Chromobacterium* species sequences available in public databases was performed to gain insight into the core and unique genes. To our knowledge, this is the first reported genome sequencing of a *C. amazonense* isolate.

## 2. Materials and Methods

### 2.1. Bacterial Isolate and DNA Extraction

*Chromobacterium* sp. strain 56AF was originally recovered in 2005 from the tropical freshwater Lake Dom Helvécio located in the Rio Doce State Park (Atlantic Rain biome), Minas Gerais, Brazil [12]. This has been a RAMSAR site since 2010 (http://www.ramsar.org) in recognition of its importance for the global conservation of biological diversity. Briefly, *Chromobacterium* sp. strain 56 AF was isolated on 25%-strength nutrient agar (Difco Laboratories, USA) at 25°C. More details were presented in a previous study of our group [12].

For the extraction of genomic DNA (gDNA), strain 56AF was grown in nutrient broth medium (Difco Laboratories, USA) at 25°C with shaking at 150 rpm for 24 h. The gDNA was extracted using the PureLink Genomic DNA kit (Thermo Fisher Scientific, USA) according to the manufacturer's instructions. gDNA quantification was performed with a Qubit® fluorometer (Thermo Fisher Scientific).

### 2.2. Phylogenetic and Phylogenomic Analyses

After the description of new *Chromobacterium* species, the phylogenetic and taxonomic position of *Chromobacterium* sp. strain 56AF was determined using 16S rRNA gene sequences (1474 bp) from 21 Neisseriaceae and Chromobacteriaceae sequences retrieved from the GenBank database (http://www.ncbi.nlm.nih.gov/). Nucleic acids were aligned using MAFFT V7 [20] with iterative refinement using the G-INS-i strategy. To optimize the dataset for phylogenetic analysis, gap-rich columns were removed from the alignment using TrimAl (Gappyout option) (version 1.3) [21] available in Phylemon [22]. The best fit model for the multiple sequence alignment was estimated using ProtTest (version 3) [23]. Altogether, 12 different evolutionary models (Blosum62, CpREV, Dayhoff, DCMut, JTT, LG, MtArt, MtMam, MtREV, RtREV, VT, and WAG) were tested with the +I, +G, and +F parameters. The evolutionary model that best fit the data was determined according to the Akaike information criterion (AIC), and support values for each node were estimated using the approximate likelihood-ratio test (aLRT).

Digital DNA-DNA hybridization (dDDH) values between the genome sequences of *Chromobacterium* sp. strain 56AF and the available *Chromobacterium* type strains (GenBank assembly accessions GCA_000711885.1, GCA_000007705.1, GCA_000971335.1, GCA_001676875.1, GCA_001953795.1, GCA_001953775.1, and GCA_001855565.1) were estimated using the online analysis tool Genome-Genome-Distance Calculator 2.1 (GGDC) [24]. The recommended distance formula 2 was taken into account to interpret the results, as it is robust against the use of incomplete genome sequences.

### 2.3. Genome Sequencing and Assembly

The genome of strain 56AF was prepared using the Nextera DNA Library Preparation Kit (Illumina Inc., USA) and sequenced using a paired-end approach on the Illumina MiSeq platform. The average insert length was ~550 bp. Reads were trimmed and filtered with Trimmomatic software (version 0.32) [25], and sequences with a Phred Q score < 22, length < 35 bp, and ambiguous bases were discarded.

Trimmed reads were assembled de novo with Velvet software (version 1.2.10) [26]. Several assemblies were computed using k-mer values from 31 to 119. Assembly results were compared, and the best assembly (k-mer 95) was selected based on the N50 and the lowest number of contigs. This was further aligned with the Mauve Multiple Genome Alignment program against the complete genome of *C. violaceum* ATCC 12472. Possible misassemblies were examined with QUAST software (version 3.0) [27] (Table S1). The genome coverage was estimated by aligning the raw reads against the assembly with BWA [28] and Samtools [29], revealing a sequence coverage of 16,482x.

### 2.4. Accession Number

This whole-genome shotgun project has been deposited at DDBJ/ENA/GenBank under the accession number MTBD00000000. The version described in this paper is the first version, MTBD01000000.

### 2.5. Genome Annotation

ORFs were predicted using the RAST server databases. Additional functional annotations were performed with the SEED database and Kyoto Encyclopedia of Genes and Genomes (KEGG). Additionally, gene discovery was performed with Prodigal software [30]. The identified ORFs were checked against prot2003-2014.fa and cog2003-2014 from the National Center for Biotechnology Information nonredundant sequence database (NCBI-Nr) through a Blastp search for the assignment of Clusters of Orthologous Group (COG) functions. tRNA and rRNA genes were identified using the Aragorn and RNAmmer software programs, respectively. The coding density of the genome was determined with Artemis software (version 16.0.0) [31] based on GenBank files generated by the RAST server. A circular genome map was generated using the BLAST Ring Image Generator (BRIG) program [32]. Phage discovery was conducted using the PHAST tool.

### 2.6. Comparative Genomic Analysis

The *C. amazonense* strain 56AF genome sequence was compared to the genomes of *C. violaceum* ATCC 12472 [17], *C. haemolyticum* DSM 19808 [33], *C. vaccinii* 21-1 [34], *C. piscinae* ND17 [35], *C. aquaticum* CC-SEYA-1 [36], *C. subtsugae* MWU 2920 [37], *C. pseudoviolaceum* LMG3953 [38], and *C. amazonense* CBMAI 310 (unpublished, GenBank assembly accession: GCA_001855565.1). The predicted proteomes were used with OrthoFinder [39] to identify orthologous genes.

## 3. Results and Discussion

**Figure 1:**
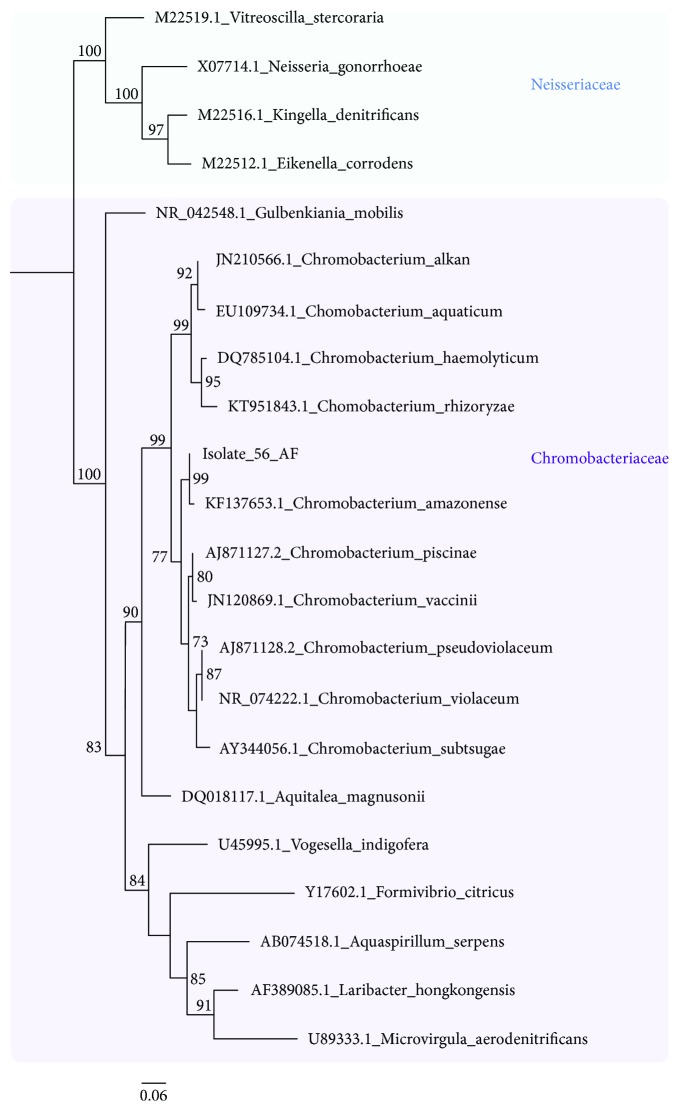
Maximum likelihood phylogenetic tree showing the evolutionary position of *C. amazonense* strain 56AF in relation to other *Chromobacterium* species. Neisseriaceae sequences were included as the outgroup. The phylogeny was reconstructed based on 22 partial 16S rRNA gene sequences and 1470 sites. Support values for each node were estimated using the approximate likelihood-ratio test (aLRT). Boxes of different colors highlight distinct families.

The phylogenetic position of *Chromobacterium* sp. strain 56AF, previously described by our group [12], was examined using the complete 16S rRNA gene sequences from 21 Neisseriaceae and Chromobacteriaceae sequences retrieved from the GenBank database. The resulting phylogeny shows the evolutionary relationships among these sequences and revealed that *Chromobacterium* sp. 56AF is closely related (94% of identity) to *C. amazonense* CBMAI 310, isolated from water samples from the Rio Negro, in the Amazon, Brazil (Figure 1). The pairwise alignment indicated that the percent identity between these two sequences is over 99.5%. In addition, the phylogenetic tree also demonstrated that the *Chromobacterium* genus forms a well-supported monophyletic clade.

The *C. amazonense* CBMAI 310 genome is 81.6% homologous with that of *Chromobacterium* sp. strain 56AF, whereas the other reference genomes exhibited dDDH values ranging from 23.10 to 42.9% when compared with the *Chromobacterium* sp. strain 56AF genome. Thus, based on the 16S rRNA gene phylogeny and dDDH data, *Chromobacterium* sp. strain 56AF has been renamed as *C. amazonense* strain 56AF.

### 3.1. General Genomic Features

**Figure 2:**
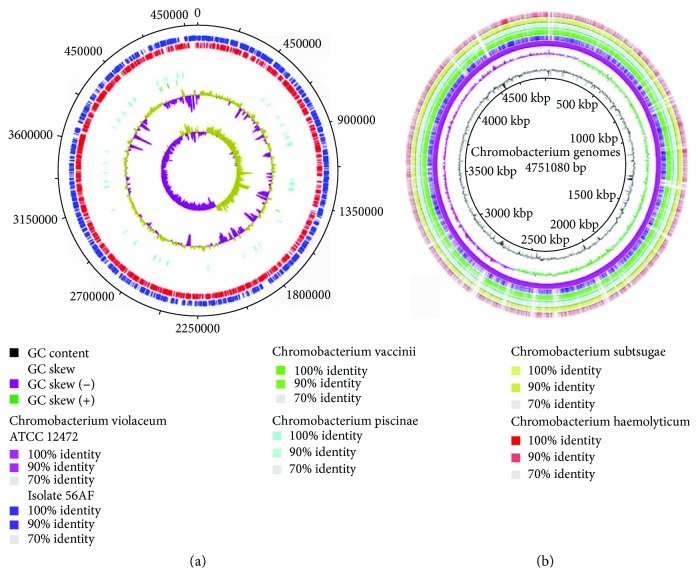
(a) Circular map of the draft genome of *C. amazonense* strain 56AF. From outer to inner circle: genes on the forward and reverse strands (predicted coding sequences colored), tRNA genes, rRNA genes, GC content, and GC skew. The circular genome map was generated using BRIG. (b**)** BLAST comparison of the draft genome of *C. amazonense* strain 56AF against the genomes of *Chromobacterium* species. The innermost ring depicts *C. violaceum* ATCC12472, followed by the query sequences of *C. amazonense* strain 56AF, *C. amazonense*, *C*. *piscinae*, *C. vaccinii*, *C*. *subtsugae*, *C. haemolyticum*, *C. pseudoviolaceum*, and *C. aquaticum*.

The *C. amazonense* strain 56AF genome is 4,556,707 bp long and distributed across 141 contigs. A map of the chromosome and the features of the genome is shown in Figure 2 and Table 1, respectively. The N50 is 169,722 bp, and the GC content is 61.95%, consistent with the values reported for *Chromobacterium* species, which range from 62.23% to 64.83%. A total of 85.8% of the final assembly was annotated as coding regions. Out of 4294 predicted coding sequences (CDSs) with an average length of 911 bp, 4205 were protein-coding genes, and 95 were RNA genes, including 88 tRNAs, six rRNAs, and one tmRNA. Among the protein-coding genes, 3298 (76.8%) were matched to functions present in the database, whereas the remaining genes were annotated as hypothetical proteins. The classification of genes into functional categories generated by RAST is shown in Table 2. Prophage sequences identified by PHAST constitute 5.8% of the bacterial chromosome, including seven intact, two incomplete, and one questionable prophage (Table S2 and Figure S1).

Among the predicted genes present in the genome of *C. amazonense* strain 56AF, we focused on virulence factors, toxins, and secretion systems, which might be of particular importance in terms of pathogenicity, survival, and adaptation to the environment.

### 3.2. Insights from the Genome Sequence

#### 3.2.1. Virulence Factors

A number of potential *Chromobacterium* virulence factors have been previously identified, including different types of secretion systems and toxins that are likely associated with *C. violaceum* and *C. haemolyticum* fatal infections in humans [17, 34]. Consistent with these reports, the draft genome of *C. amazonense* strain 56AF includes CDSs with significant similarity to virulence factors (Table S3) that were previously described for strains belonging to the *Chromobacterium* genus among others, as discussed in the following sections.

#### 3.2.2. Secretion Systems

There are five well-studied double-membrane-spanning secretion systems (T1SS, T2SS, T3SS, T4SS, and T6SS) present in a wide variety of pathogenic and nonpathogenic gram-negative bacteria. These systems secrete products with diverse functions, such as those involved in adhesion, pathogenicity, adaptation, and survival [40]. Here, we identified a number of particularly interesting proteins that are part of the T1SS, T2SS, T3SS, T4SS, and T6SS machinery.

In gram-negative bacteria, T1SS is composed of three membrane proteins, two in the inner membrane belonging to the ABC transporter and membrane fusion protein (MFP) families and a third porin-like protein in the outer membrane [41], known as LapB, LapC, and TolC, respectively. The products secreted by T1SS are diverse in size and function and frequently associated with nutrient acquisition (lipases and proteases) and virulence, for example, colicin V, which was found in the *C. amazonense* strain 56AF genome.

T2SS consists of a large multiprotein machinery with 12–15 components known as general secretion pathway (Gsp) proteins [40]. Analysis of the *C. amazonense* strain 56AF genome revealed the presence of all of these proteins with the exception of GspC, which may be missing from the genome assembly. An additional T2SS protein, GspN, was identified; however, its function and location are unclear [42].

T3SS is found in a large number of gram-negative pathogens and symbionts [43]. This secretion system is composed of structural, translocator, and effector proteins. Similar to *C. violaceum* ATCC 12472, *C. amazonense* strain 56AF possesses the *Chromobacterium* pathogenicity islands (Cpi) 1, 1a, and 2, whereas *C. haemolyticum* T124 lacks the Cpi-2-like second T3SS. These data suggest the potential virulence of *C. amazonense* strain 56AF.

T4SS is found in both gram-negative and gram-positive bacteria and in some archaea and allows the translocation of DNA and proteins into target cells, playing an important role in the pathogenesis of a wide range of bacteria [44]. Among the secretion systems, T4SS is unique for its ability to mediate the conjugation of plasmid DNA [40]. It consists of 12 proteins (VirB1–VirB11 and VirD4), of which only VirB3 and VirB7 were missing from the *C. amazonense* strain 56AF genome assembly. This is the first report of T4SS in the *Chromobacterium* genus, which is considered to be critical for pathogenicity.

Finally, T6SS is broadly distributed among Proteobacteria. It translocates toxic effector proteins into eukaryotic and prokaryotic cells and has an important role in pathogenesis, bacterial communication, and interactions with the environment [45–47]. T6SS consists of a 13-protein core and a set of conserved accessory proteins [48]. In the genome of *C. amazonense* strain 56AF, 7 out of the 13 T6SS-associated proteins (ImpA, ImpB, ImpC, ImpF, ImpG, ImpH, and ImpJ) were found, in addition to the T4SS homolog IcmF. It is also known that T6SS secretes hemolysin and the effector protein VrgG, both of which are present in the *C. amazonense* strain 56AF genome.

#### 3.2.3. Toxins

Toxins can be identified by the presence of a variety of proteins, such as hemolysins, hemolytic enterotoxins, colicin V, lytic proteins, and Nudix hydrolases. These toxins are associated with pathogenic bacteria [49–52] and are present in the genome of *C. amazonense* strain 56AF.

Important virulence factors for host cell invasion were found, for example, CDSs encoding a hydrolase cell-wall associated protein, metalloproteases, lypolitic proteins, collagenases, and the proteolytic enzymes KpSS and KpSC. The last two are conserved proteins involved in capsular polysaccharides assembly systems [53], suggesting the potential of *C. amazonense* strain 56AF to synthesize capsular polysaccharides, which can act as important virulence factors in many pathogenic bacteria [53]. Moreover, genes involved in flagellar biosynthesis were identified. Flagella and swimming motility are present in all the genera of the family Chromobacteriaceae [1] and are considered to be important contributors to host colonization for most pathogens.

Unlike other *Chromobacterium* species, the genome of *C. amazonense* strain 56AF contains the gene for pore-forming toxin alpha-hemolysin *(hlyA)*, which shares homology with the gene in *Vibrio cholerae*. It should be noted that hemolysin is a well-known virulence factor, and hemolytic activity has been detected in other *Chromobacterium* species, such as *C. violaceum*, *C. haemolyticum*, *C. aquaticum*, and *C. subtsugae* [5, 17, 54]. Thus, some *Chromobacterium* species have the potential to produce different cytolytic toxins that may contribute to their pathogenicity.

A set of genes with potential roles in multiple antibiotic resistance were identified in the *C. amazonense* strain 56AF genome and included CDSs encoding the RND efflux transporter, tripartite multidrug resistance system, Bcr/CflA family drug resistance transporter, multiple antibiotic resistance MarC protein, and resistance to *β*-lactams. Some efflux pumps not only confer clinically relevant resistance to antibiotics but also have a role in bacterial pathogenicity and may be beneficial for bacterial survival [55]. Regarding *β*-lactams, *C. amazonense* strain 56AF genome presented CDSs related to *β*-lactamases, including extended spectrum *β*-lactamases Ambler class C *β*-lactamase, penicillinases, metallo-*β*-lactamase, and metal-dependent hydrolases of the *β* -lactamase superfamily I PhnP protein. It is worth mentioning that they hydrolyze almost all clinically used *β*-lactams including carbapenems, thereby representing a therapeutic challenge. These results were supported by antimicrobial susceptibility testing obtained by Lima-Bittencourt et al. [12] for *Chromobacterium* sp. strain 56AF, which found resistance to ampicillin, ampicillin/clavulanic acid, and cefotaxime (third-generation cephalosporin), widely used for the treatment of bacterial infections. Other *Chromobacterium* species also exhibit *β*-lactamases, such as *C. piscinae* and *C. haemolyticum* [56]. Other antibiotic resistance genes encode resistance to bicyclomycin, fosfomycin, mitomycin, polymyxin (PmrJ, ArnC, and ArnT proteins), and fosmidomycin. Importantly, the genome contains genes related to resistance to several toxic compounds, including tellurite, cobalt, zinc-cadmium (CzcA), and fusaric acid, a mycotoxin produced by several *Fusarium* species that cause diseases in economically important crops [57].

Additionally, the annotation of the *C. amazonense* strain 56AF revealed its potential to produce esterases, lipase, auxins, chitinases, phytoene synthase and phytoene desaturase, polyhydroxyalkanoates, glutathione S-transferases, and violacein, suggesting that this strain might have potential for biotechnological purposes. The genome contains predicted toxin-antitoxin genes, in particular those of *hig*AB and *doc*/*phd*, involved in plasmid stabilization [58], as well as the antitoxin YgiT (MqsA), which helps to mediate the bacterial general stress response [59], although the gene encoding MqsR toxin was absent. Finally, plastocyanin/azurin genes were also found, suggesting that this strain may participate in denitrifying processes.

### 3.3. Comparison of Eight *Chromobacterium* Genomes

**Figure 3:**
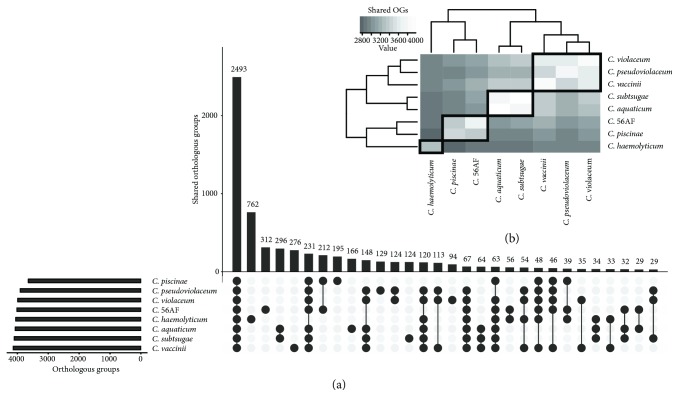
Distribution of orthologous groups (OGs) among the genomes of *C. amazonense*, *C*. *piscinae*, C. *pseudoviolaceum*, *C. violaceum*, *C. amazonense* strain 56AF, *C. haemolyticum*, C. *aquaticum*, *C*. *subtsugae*, and *C. vaccinii*. (a) The bars represent the number of unique or shared OGs among *Chromobacterium* genomes marked with the filled circle. (b) The heat map shows the number of shared OGs between different *Chromobacterium* species. The dendrogram is based on Euclidean distance of the shared OG matrix.

Based on orthologous groups (OGs) analysis, the eight *Chromobacterium* genomes examined contain a total of 7220 OGs, with a conserved core genome of 2467 OGs present in all species (Figure 3). A large fraction of the flexible genes (65.8%), that is, those present in one or more but not all of the genomes, was mapped, among which 2069 were species-specific and 2684 were shared noncore. These findings are consistent with the great metabolic flexibility of *Chromobacterium*, conferring to their members a possible selective advantage to colonize and to survive in diverse ecosystems. It should be noted that *C. violaceum* contained the largest number of OGs shared with other *Chromobacterium* genomes. Comparisons of *C. amazonense* strain 56AF with the seven other genomes revealed a subset of OGs (301) that were unique to *C. amazonense* strain 56AF, and these may contribute to the species-specific features of *C. amazonense*. The unique set of *C. amazonense* strain 56AF genes was associated with the COG functional categories carbohydrate transport and metabolism (G); cell cycle control, cell division, and chromosome partitioning (D); amino acid transport and metabolism (E); nucleotide transport and metabolism (F); replication, recombination, and repair (L); defense mechanisms (V); and cell motility (N). Considering that seven out of the eight genomes analyzed here are draft sequences (all except for *C. violaceum*), it is likely that individual genes are missing from these assemblies.

## 4. Conclusions

*C. amazonense* strain 56AF is the first strain of the genus known to contain predicted genes associated with five of the six double-membrane-spanning secretion systems (types 1–4 and 6) present in gram-negative bacteria, of which T4SS has not been reported in any other sequenced *Chromobacterium*. Additionally, the presence of several predicted genes encoding potential multidrug resistance and detoxification compounds suggests that this isolate harbors a genomic repertoire capable of dealing with environmental toxins, which may be beneficial to its adaptation and survival. Moreover, *C. amazonense* strain 56AF contains a number of relevant virulence-related genes, suggesting its potential pathogenicity. In summary, our findings confirm the versatility, adaptability, and biotechnological potential of this bacterium. Moreover, our results provide important insights into the genomics of *Chromobacterium* species, leading to a better understanding of the biology of this genus. Further functional studies will contribute with new information about its pathogenic potential.

## Figures and Tables

**Table 1:** Comparison of the genomic properties of *Chromobacterium* spp.

*Chromobacterium* spp.	Size (Mb)	Contig	GC (%)	Gene	rRNA	tRNA	Other RNA	Reference
*C. amazonense* 56AF	4,56	141	62	4,294	8	89	1	This study
*C. violaceum* ATCC 12472	4,75	1	64.8	4,438	25	98	5	[32]
*C. haemolyticum* DSM 19808	5,03	67	62.8	4,595	13	57	4	[33]
*C. piscinae* ND 17	4,09	223	62.6	3,916	4	81	1	[35]
*C. subtsugae* MWU 2920	4,67	152	64.9	4,313		77	4	[37]
C. *vaccinii* MWU 205	4,97	152	64.44	4463	6	74	4	[34]
*C. aquaticum* CC-SEYA-1	4.77	1,63	64.8	4492		33	4	[36]
*C. pseudoviolaceum* LMG 3953	4.63	326	64.7	4404		33	5	[12]

**Table 2:** SEED subsystems distribution of the *Chromobacterium amazonense* strain 56AF genome based on MG-RAST annotation.

Subsystem category	Subsystem feature counts
Sulfur metabolism	32
Phosphorus metabolism	47
Carbohydrates	242
Amino acids and derivatives	417
Fatty acids, lipid, and isoprenoids	103
Metabolism secondary	4
Nitrogen metabolism	13
Metabolism of aromatic compounds	24
Cofactors, vitamins, prosthetic groups, and pigments	242
Nucleosides and nucleotides	90
Dormancy and sporulation	1
Respiration	133
Stress response	134
DNA metabolism	99
Membrane transport	156
Regulation and cell signaling	88
Motility and chemotaxis	176
Cell division and cell cycle	35
Protein metabolism	298
RNA metabolism	135
Iron acquisition and metabolism	34
Phages, prophages, transposable elements and plasmids	65
Miscellaneous	45
Photosynthesis	0
Potassium metabolism	20
Virulence, disease, and defense	88
Cell wall and capsule	166

## Abbreviations

ORFs: Open reading frames
gDNA: Genomic DNA
dDDH: Digital DNA-DNA hybridization
KEGG: Kyoto Encyclopedia of Genes and Genomes
COGs: Clusters of Orthologous Groups
OGs: Orthologous groups
TSS: Type secretion system.

## Supplementary Materials

Supplementary Materials Table S1: misassembly report based on the alignment of *C. amazonense* strain 56AF, extracted from QUAST. Table S2: phages present in the genome of *C. amazonense* strain 56AF. Table S3: virulence factors identified in the genome of *C. amazonense* strain 56AF according to the virulence factor database of pathogenic bacteria. Figure S1: phages present in the genome of *C. amazonense* strain 56AF. Circular view extracted from PHAST.
