# Supplementary material for: Insights into the Genome Sequence of Chromobacterium amazonense Isolated from a Tropical Freshwater Lake
Source: Int J Genomics. 2018 May 20;2018:1062716. doi: 10.1155/2018/1062716 (PMC5985088; doi:10.1155/2018/1062716)
Supplement: Supplementary Materials — Table S1: misassembly report based on the alignment of C. amazonense strain 56AF, extracted from QUAST. Table S2: phages present in the genome of C. amazonense strain 56AF. Table S3: virulence factors identified in the genome of C. amazonense strain 56AF according to the virulence factor database of pathogenic bacteria. Figure S1: phages present in the genome of C. amazonense strain 56AF. Circular view extracted from PHAST. [file 1062716.f1.pdf]

Table S1. Misassembly report based on the alignment of *C. amazonense* strain 56AF, extracted from QUAST.

Misassemblies	0
Relocations	0
Translocations	0
Inversions	0
Misassembled contigs	0
Misassembled contig length	0
Local misassemblies	1
Scaffold gap size misassemblies	0
Indels	1194
Short indels	59
Long indels	59
Indels length	74

Table S2. Phages present in the genome of *C. amazonense* strain 56AF.

Size Kb)	Completness	Possible phage	CDS (n)	Score	Location	Access number
22.3	Intact	Mannhe_VB_MhM	25	140	223399-245719	NC028766
24	Questionable	Burkho_BcepMU	27	70	298005-322006	NC005882
42.9	Intact	Ralsto RSY1	38	100	9932300-1035214	NC025115
24.1	Intact	BurKho KS9	26	140	1175203-1199347	NC013055
6.5	Incomplete	Entero 186	7	60	1233457-1240011	NC001317
31.5	Intact	Ralsto RS 138	33	120	1853482-1885064	NC029107
10.6	Intact	Stx2 converting	11	110	2058319-2068998	NC011357
18.1	Intact	Ralsto RSB2	19	100	2394605-2412770	NC023736
12.9	Incomplete	Escher HK 639	20	20	4266813-4279731	NC016158
75.5	Intact	Shigel sfIV	109	150	4304439-4379944	NC022749

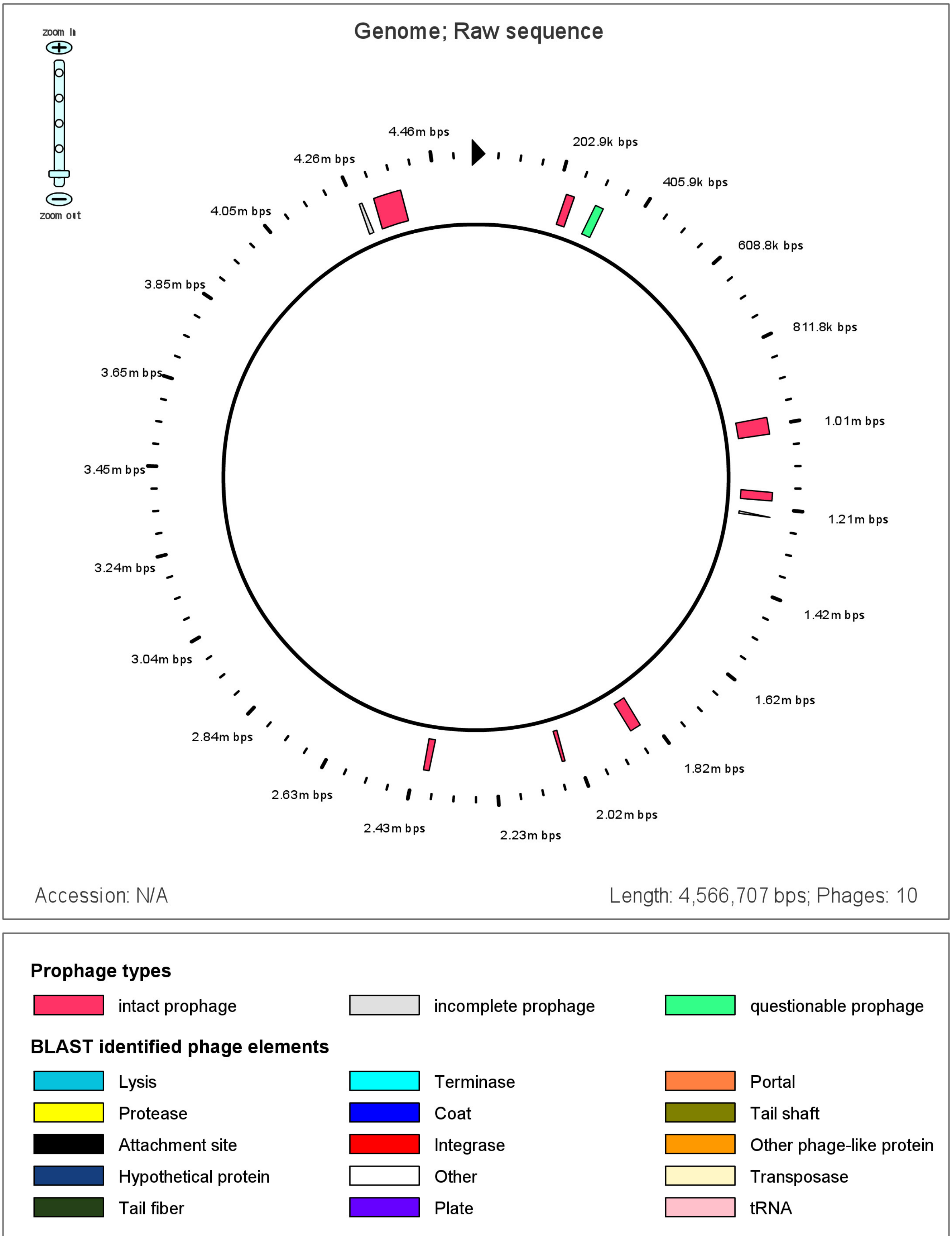
zoom in
zoom out
Genome; Raw sequence
4.46m bps
202.9k bps
405.9k bps
608.8k bps
811.8k bps
1.01m bps
1.21m bps
1.42m bps
1.62m bps
1.82m bps
2.02m bps
2.23m bps
2.43m bps
2.63m bps
2.84m bps
3.04m bps
3.24m bps
3.45m bps
3.65m bps
3.85m bps
4.05m bps
4.26m bps
Accession: N/A
Length: 4,566,707 bps; Phages: 10
Prophage types
intact prophage
incomplete prophage
questionable prophage
BLAST identified phage elements
Lysis
Terminase
Portal
Protease
Coat
Tail shaft
Attachment site
Integrase
Other phage-like protein
Hypothetical protein
Other
Transposase
Tail fiber
Plate
tRNA

VFDB accession number	Gene product description	*Chromobacterium amazonense* 56AF predicted gene
VFG000031(gb|NP_878988)	(bplH) putative glycosyl transferase [LPS (VF0033)] [Bordetella pertussis Tohama I]	c_amazonense.gapfilled_200_mauve.fasta_3615
VFG000034(gb|NP_878991)	(bplE) probable glycosyl transferase [LPS (VF0033)] [Bordetella pertussis Tohama I]	c_amazonense.gapfilled_200_mauve.fasta_3613
VFG000048(gb|NP_880888)	(bscN) ATP synthase in type III secretion system [TTSS (VF0035)] [Bordetella pertussis Tohama I]	c_amazonense.gapfilled_200_mauve.fasta_547
VFG000077(gb|NP_465991)	(clpP) ATP-dependent Clp protease proteolytic subunit [ClpP (VF0074)] [Listeria monocytogenes EGD-e]	c_amazonense.gapfilled_200_mauve.fasta_1612
VFG000079(gb|NP_463763)	(clpC) endopeptidase Clp ATP-binding chain C [ClpC (VF0072)] [Listeria monocytogenes EGD-e]	c_amazonense.gapfilled_200_mauve.fasta_3253
VFG000099(gb|NP_230484)	(tcpE) toxin co-regulated pilus biosynthesis protein E [TCP (VF0126)] [Vibrio cholerae O1 biovar El Tor str. N16961]	c_amazonense.gapfilled_200_mauve.fasta_4213
VFG000104(gb|NP_230488)	(acfB) accessory colonization factor AcfB [ACF (VF0127)] [Vibrio cholerae O1 biovar El Tor str. N16961]	c_amazonense.gapfilled_200_mauve.fasta_744
VFG000112(gb|NP_253216)	(pilB) type 4 fimbrial biogenesis protein PilB [Type IV pili (VF0082)] [Pseudomonas aeruginosa PAO1]	c_amazonense.gapfilled_200_mauve.fasta_3405
VFG000119(gb|NP_253948)	(algR) alginate biosynthesis regulatory protein AlgR [Alginate (VF0091)] [Pseudomonas aeruginosa PAO1]	c_amazonense.gapfilled_200_mauve.fasta_1262
VFG000121(gb|NP_249453)	(algU) alginate biosynthesis protein AlgZ/FimS [Alginate (VF0091)] [Pseudomonas aeruginosa PAO1]	c_amazonense.gapfilled_200_mauve.fasta_2172
VFG000151(gi:15596627)	(lasR) transcriptional regulator LasR [Quorum sensing (VF0093)] [Pseudomonas aeruginosa PAO1]	c_amazonense.gapfilled_200_mauve.fasta_268
VFG000259(gb|NP_273145)	(lipA) capsule polysaccharide modification protein LipA [Capsule (VF0079)] [Neisseria meningitidis MC58]	c_amazonense.gapfilled_200_mauve.fasta_429
VFG000313(gb|NP_207965)	(gluP) glucose/galactose transporter [LPS (VF0056)] [Helicobacter pylori 26695]	c_amazonense.gapfilled_200_mauve.fasta_478
VFG000322(gb|NP_206846)	(wbcJ) GDP fucose synthase [LPS (VF0056)] [Helicobacter pylori 26695]	c_amazonense.gapfilled_200_mauve.fasta_3531
VFG000477(gi:16766230)	(rpoS) sigma S (sigma 38) factor of RNA polymerase, major sigmafactor during stationary phase [RpoS (VF0112)] [Salmonella enterica subsp. enterica serovar Typhimurium str. LT2]	c_amazonense.gapfilled_200_mauve.fasta_3265
VFG000478(gi:16764063)	(fur) transcriptional repressor of iron-responsive genes (Fur family) (ferric uptake regulator) [Fur (VF0113)] [Salmonella enterica subsp. enterica serovar Typhimurium str. LT2]	c_amazonense.gapfilled_200_mauve.fasta_2732
VFG000670(gb|NP_706258)	(gtrB) bactoprenol glucosyl transferase [LPS (VF0124)] [Shigella flexneri 2a str. 301]	c_amazonense.gapfilled_200_mauve.fasta_1081
VFG000670(gb|NP_706258)	(gtrB) bactoprenol glucosyl transferase [LPS (VF0124)] [Shigella flexneri 2a str. 301]	c_amazonense.gapfilled_200_mauve.fasta_605
VFG000700(gb|AAP42192)	(bsc1) bifunctional ribulose 5-phosphate reductase CDP-ribitol pyrophosphorylase [Capsule (VF0043)] [Haemophilus influenzae str. 1007]	c_amazonense.gapfilled_200_mauve.fasta_3162
VFG000705(gb|AAP42197)	(hscB) capsular polysaccharide export protein HscB [Capsule (VF0043)] [Haemophilus influenzae str. 1007]	c_amazonense.gapfilled_200_mauve.fasta_428
VFG000751(gb|YP_002332159)	(bfpB) BfpB secretin [BFP (VF0174)] [Escherichia coli O127:H6 str. E2348/69]	c_amazonense.gapfilled_200_mauve.fasta_1864
VFG000752(gb|YP_002332160)	(bfpC) BfpC, bitopic inner membrane protein [BFP (VF0174)] [Escherichia coli O127:H6 str. E2348/69]	c_amazonense.gapfilled_200_mauve.fasta_1863
VFG000755(gb|YP_002332163)	(bfpE) BfpE, polytopic inner membrane protein [BFP (VF0174)] [Escherichia coli O127:H6 str. E2348/69]	c_amazonense.gapfilled_200_mauve.fasta_1860
VFG000756(gb|BAA84845)	(bfpF) retraction ATPase BfpF [BFP (VF0174)] [Escherichia coli B171]	c_amazonense.gapfilled_200_mauve.fasta_1859
VFG000930(gb|NP_752604)	(entF) enterobactin synthase multienzyme complex component, ATP-dependent [Enterobactin (VF0228)] [Escherichia coli CFT073]	c_amazonense.gapfilled_200_mauve.fasta_1314
VFG001214(gb|NP_253237)	(pilR) two-component response regulator PilR [Type IV pili (VF0082)] [Pseudomonas aeruginosa PAO1]	c_amazonense.gapfilled_200_mauve.fasta_3762
VFG001214(gb|NP_253237)	(pilR) two-component response regulator PilR [Type IV pili (VF0082)] [Pseudomonas aeruginosa PAO1]	c_amazonense.gapfilled_200_mauve.fasta_3924
VFG001214(gb|NP_253237)	(pilR) two-component response regulator PilR [Type IV pili (VF0082)] [Pseudomonas aeruginosa PAO1]	c_amazonense.gapfilled_200_mauve.fasta_906
VFG001269(gb|NP_879579)	(cyaB) cyclolysin secretion ATP-binding protein [Cya (VF0028)] [Bordetella pertussis Tohama I]	c_amazonense.gapfilled_200_mauve.fasta_1926
VFG001269(gb|NP_879579)	(cyaB) cyclolysin secretion ATP-binding protein [Cya (VF0028)] [Bordetella pertussis Tohama I]	c_amazonense.gapfilled_200_mauve.fasta_517
VFG001448(gb|AAA21682)	(kpsD) KpsD [K1 capsule (VF0239)] [Escherichia coli O18:K1:H7 str. RS218]	c_amazonense.gapfilled_200_mauve.fasta_436
VFG001449(gb|AAA24047)	(kpsT) KpsT [K1 capsule (VF0239)] [Escherichia coli O18:K1:H7 str. RS218]	c_amazonense.gapfilled_200_mauve.fasta_438
VFG001450(gb|AAA24046)	(kpsM) KpsM [K1 capsule (VF0239)] [Escherichia coli O18:K1:H7 str. RS218]	c_amazonense.gapfilled_200_mauve.fasta_439
VFG001857(gb|YP_094782)	(iraB) Di/tripeptide permease [IraAB (VF0293)] [Legionella pneumophila subsp. pneumophila str. Philadelphia 1]	c_amazonense.gapfilled_200_mauve.fasta_3332
VFG001861(gb|YP_094248)	(katA) catalase/(hydro)peroxidase [KatAB (VF0168)] [Legionella pneumophila subsp. pneumophila str. Philadelphia 1]	c_amazonense.gapfilled_200_mauve.fasta_2372
VFG001867(gb|YP_096960)	(sodB) superoxide dismutase [SodB (VF0169)] [Legionella pneumophila subsp. pneumophila str. Philadelphia 1]	c_amazonense.gapfilled_200_mauve.fasta_1807
VFG001965(gb|NP_282578)	(Cj1437c) aminotransferase [Capsule (VF0323)] [Campylobacter jejuni subsp. jejuni NCTC 11168]	c_amazonense.gapfilled_200_mauve.fasta_500
VFG001990(gb|YP_094942)	(flgA) flagellar basal body P-ring biosynthesis protein FlgA [Flagella (VF0157)] [Legionella pneumophila subsp. pneumophila str. Philadelphia 1]	c_amazonense.gapfilled_200_mauve.fasta_883
VFG002218(gb|NP_541012)	(virB11) type IV secretion system protein ATPase VirB11 [VirB type IV secretion system (VF0365)] [Brucella melitensis bv. 1 str. 16M]	c_amazonense.gapfilled_200_mauve.fasta_4204
VFG002268(gb|YP_034271)	(trwE) Trw type IV secretion sysmtem VirB10 homolog, trwE protein [Trw type IV secretion system (VF0372)] [Bartonella henselae str. Houston-1]	c_amazonense.gapfilled_200_mauve.fasta_4205
VFG002420(gb|NP_644838)	(adsA) Adenosine synthase A [AdsA (VF0422)] [Staphylococcus aureus subsp. aureus MW2]	c_amazonense.gapfilled_200_mauve.fasta_2516
VFG002420(gb|NP_644838)	(adsA) Adenosine synthase A [AdsA (VF0422)] [Staphylococcus aureus subsp. aureus MW2]	c_amazonense.gapfilled_200_mauve.fasta_2776
VFG002501(gb|YP_106857)	(fliG) flagellar motor switch protein G [Flagella (VF0430)] [Burkholderia pseudomallei K96243]	c_amazonense.gapfilled_200_mauve.fasta_2566
VFG002519(gb|YP_109887)	(fliA) flagellar biosynthesis sigma factor [Flagella (VF0430)] [Burkholderia pseudomallei K96243]	c_amazonense.gapfilled_200_mauve.fasta_913
VFG002550(gb|YP_109386)	(wcbP) capsular polysaccharide biosynthesis dehydrogenase/reductase [Capsule I (VF0436)] [Burkholderia pseudomallei K96243]	c_amazonense.gapfilled_200_mauve.fasta_582
VFG002679(gi:16122052)	(flgC) flagellar basal body rod protein [Flagella (cluster I) (CVF039)] [Yersinia pestis CO92]	c_amazonense.gapfilled_200_mauve.fasta_2360
VFG002705(gi:16123281)	(ddhD) CDP-6-deoxy-delta-3,4-glucoseen reductase [O-antigen (CVF043)] [Yersinia pestis CO92]	c_amazonense.gapfilled_200_mauve.fasta_3361
VFG002901(gi:108808062)	(YPA_2067) histidine transport system permease protein HisQ [O-antigen (CVF043)] [Yersinia pestis Antiqua]	c_amazonense.gapfilled_200_mauve.fasta_2422
VFG002901(gi:108808062)	(YPA_2067) histidine transport system permease protein HisQ [O-antigen (CVF043)] [Yersinia pestis Antiqua]	c_amazonense.gapfilled_200_mauve.fasta_2519
VFG002901(gi:108808062)	(YPA_2067) histidine transport system permease protein HisQ [O-antigen (CVF043)] [Yersinia pestis Antiqua]	c_amazonense.gapfilled_200_mauve.fasta_2520
VFG002901(gi:108808062)	(YPA_2067) histidine transport system permease protein HisQ [O-antigen (CVF043)] [Yersinia pestis Antiqua]	c_amazonense.gapfilled_200_mauve.fasta_3148
VFG002901(gi:108808062)	(YPA_2067) histidine transport system permease protein HisQ [O-antigen (CVF043)] [Yersinia pestis Antiqua]	c_amazonense.gapfilled_200_mauve.fasta_3550
VFG002901(gi:108808062)	(YPA_2067) histidine transport system permease protein HisQ [O-antigen (CVF043)] [Yersinia pestis Antiqua]	c_amazonense.gapfilled_200_mauve.fasta_712
VFG002901(gi:108808062)	(YPA_2067) histidine transport system permease protein HisQ [O-antigen (CVF043)] [Yersinia pestis Antiqua]	c_amazonense.gapfilled_200_mauve.fasta_713
VFG002901(gi:108808062)	(YPA_2067) histidine transport system permease protein HisQ [O-antigen (CVF043)] [Yersinia pestis Antiqua]	c_amazonense.gapfilled_200_mauve.fasta_97
VFG003393(gi:51595350)	(ddhB) CDP-glucose 4,6-dehydratase [O-antigen (CVF043)] [Yersinia pseudotuberculosis IP 32953]	c_amazonense.gapfilled_200_mauve.fasta_3451
VFG003394(gi:51595351)	(ddhC) putative CDP-4-keto-6-deoxy-D-glucose-3-dehydratase [O-antigen (CVF043)] [Yersinia pseudotuberculosis IP 32953]	c_amazonense.gapfilled_200_mauve.fasta_3532
VFG003413(gi:51596047)	(fliS) flagellar protein FliS [Flagella (cluster I) (CVF039)] [Yersinia pseudotuberculosis IP 32953]	c_amazonense.gapfilled_200_mauve.fasta_3433
VFG003433(gi:51596013)	(flgG) flagellar basal-body rod protein FlgG (distal rod protein) [Flagella (cluster I) (CVF039)] [Yersinia pseudotuberculosis IP 32953]	c_amazonense.gapfilled_200_mauve.fasta_2356
VFG003636(gi:16761645)	(orgC) hypothetical protein found within S. typhi pathogenicity island 1 [TTSS (SPI-1 encode) (CVF031)] [Salmonella enterica subsp. enterica serovar Typhi str. CT18]	c_amazonense.gapfilled_200_mauve.fasta_1717
VFG003668(gi:56414826)	(prgH) pathogenicity 1 island effector protein [TTSS (SPI-1 encode) (CVF031)] [Salmonella enterica subsp. enterica serovar Paratyphi A str. ATCC 9150]	c_amazonense.gapfilled_200_mauve.fasta_1722
VFG003728(gi:56414843)	(spaO) type III secretion protein [TTSS (SPI-1 encode) (CVF031)] [Salmonella enterica subsp. enterica serovar Paratyphi A str. ATCC 9150]	c_amazonense.gapfilled_200_mauve.fasta_1848
VFG004096(gi:16759521)	(fimZ) probable transcriptional regulator (FimXZ protein) [Fim (CVF003)] [Salmonella enterica subsp. enterica serovar Typhi str. CT18]	c_amazonense.gapfilled_200_mauve.fasta_2037
VFG004379(gi:16767813)	(stjB) putative fimbrial usher protein [Stj (AI067)] [Salmonella enterica subsp. enterica serovar Typhimurium str. LT2]	c_amazonense.gapfilled_200_mauve.fasta_1324
VFG005021(gi:73661373)	(SSP0064) putative glycosyltransferase [Capsule (CVF110)] [Staphylococcus saprophyticus subsp. saprophyticus ATCC 15305]	c_amazonense.gapfilled_200_mauve.fasta_3536
VFG005341(gi:15674453)	(plr/gapA) glyceraldehyde-3-phosphate dehydrogenase [Streptococcal plasmin receptor/GAPDH (CVF123)] [Streptococcus pyogenes M1 GAS]	c_amazonense.gapfilled_200_mauve.fasta_84
VFG005356(gi:116516442)	(plr/gapA) glyceraldehyde-3-phosphate dehydrogenase, type I [Streptococcal plasmin receptor/GAPDH (CVF123)] [Streptococcus pneumoniae D39]	c_amazonense.gapfilled_200_mauve.fasta_448
VFG005535(gi:71911425)	(tig/ropA) trigger factor [Trigger factor (CVF149)] [Streptococcus pyogenes MGAS5005]	c_amazonense.gapfilled_200_mauve.fasta_1611
VFG005582(gi:125717729)	(eno) Enolase, putative [Streptococcal enolase (CVF153)] [Streptococcus sanguinis SK36]	c_amazonense.gapfilled_200_mauve.fasta_3010
VFG005767(gb|NP_687682)	(cylG) 3-ketoacyl-ACP-reductase CylG [Beta-hemolysin/cytolysin (CVF171)] [Streptococcus agalactiae 2603V/R]	c_amazonense.gapfilled_200_mauve.fasta_2964
VFG005997(gi:24379858)	(rmlA) putative glucose-1-phosphate thymidyltransferase [Capsule (CVF186)] [Streptococcus mutans UA159]	c_amazonense.gapfilled_200_mauve.fasta_3521
VFG006201(gi:59800790)	(pilT2) putative twitching motility-like protein [GC pili (AI096)] [Neisseria gonorrhoeae FA 1090]	c_amazonense.gapfilled_200_mauve.fasta_75
VFG006242(gi:121635698)	(porB) porin, major outer membrane protein P.I [PorB (CVF195)] [Neisseria meningitidis FAM18]	c_amazonense.gapfilled_200_mauve.fasta_1512
VFG006242(gi:121635698)	(porB) porin, major outer membrane protein P.I [PorB (CVF195)] [Neisseria meningitidis FAM18]	c_amazonense.gapfilled_200_mauve.fasta_2535
VFG006255(gi:59800669)	(fbpB) ABC transporter, permease protein, iron related [ABC transporter (CVF197)] [Neisseria gonorrhoeae FA 1090]	c_amazonense.gapfilled_200_mauve.fasta_2581
VFG006259(gi:59800668)	(fbpC) ABC transporter, ATP-binding protein, iron related [ABC transporter (CVF197)] [Neisseria gonorrhoeae FA 1090]	c_amazonense.gapfilled_200_mauve.fasta_2421
VFG006259(gi:59800668)	(fbpC) ABC transporter, ATP-binding protein, iron related [ABC transporter (CVF197)] [Neisseria gonorrhoeae FA 1090]	c_amazonense.gapfilled_200_mauve.fasta_336
VFG006259(gi:59800668)	(fbpC) ABC transporter, ATP-binding protein, iron related [ABC transporter (CVF197)] [Neisseria gonorrhoeae FA 1090]	c_amazonense.gapfilled_200_mauve.fasta_98
VFG006288(gi:15794866)	(exbB) biopolymer transport protein [Ton system (CVF202)] [Neisseria meningitidis Z2491]	c_amazonense.gapfilled_200_mauve.fasta_290
VFG006295(gi:59801725)	(exbD) ExbD [Ton system (CVF202)] [Neisseria gonorrhoeae FA 1090]	c_amazonense.gapfilled_200_mauve.fasta_3893
VFG006392(gi:15611540)	(cag5) cag island protein, DNA transfer protein [Cag PAI type IV secretion system (CVF217)] [Helicobacter pylori J99]	c_amazonense.gapfilled_200_mauve.fasta_4202
VFG006719(gi:16800743)	(lap) hypothetical protein [Listeria adhesion protein (CVF228)] [Listeria innocua Clip11262]	c_amazonense.gapfilled_200_mauve.fasta_1063
VFG006813(gi:116873279)	(lspA) lipoprotein signal peptidase [Lipoprotein-specific signal peptidase II (CVF249)] [Listeria welshimeri serovar 6b str. SLCC5334]	c_amazonense.gapfilled_200_mauve.fasta_3093
VFG006892(gi:147673778)	(acfB) accessory colonization factor AcfB [Accessory colonization factor (CVF257)] [Vibrio cholerae O395]	c_amazonense.gapfilled_200_mauve.fasta_1009
VFG006948(gi:37681131)	(mshL) MSHA biogenesis protein MshL [Mannose-sensitive hemagglutinin (MSHA type IV pilus) (CVF259)] [Vibrio vulnificus YJ016]	c_amazonense.gapfilled_200_mauve.fasta_4274
VFG006967(gi:59710969)	(mshE) type 4 fimbrial assembly protein PilB [Mannose-sensitive hemagglutinin (MSHA type IV pilus) (CVF259)] [Vibrio fischeri ES114]	c_amazonense.gapfilled_200_mauve.fasta_4277
VFG007025(gi:27366916)	(rtxB) ABC-type bacteriocin/lantibiotic exporter [RTX toxin (CVF263)] [Vibrio vulnificus CMCP6]	c_amazonense.gapfilled_200_mauve.fasta_3567
VFG007026(gi:37676694)	(rtxB) RTX toxin transporter [RTX toxin (CVF263)] [Vibrio vulnificus YJ016]	c_amazonense.gapfilled_200_mauve.fasta_516
VFG007033(gi:15600987)	(tlh) thermolabile hemolysin [Thermolabile hemolysin (CVF265)] [Vibrio cholerae O1 biovar El Tor str. N16961]	c_amazonense.gapfilled_200_mauve.fasta_242
VFG007224(gi:15642208)	(viuB) vibriobactin utilization protein ViuB [Vibriobactin utilization (CVF275)] [Vibrio cholerae O1 biovar El Tor str. N16961]	c_amazonense.gapfilled_200_mauve.fasta_1765
VFG007406(gi:15642121)	(fliQ) flagellar biosynthesis protein [Flagella (VF0519)] [Vibrio cholerae O1 biovar El Tor str. N16961]	c_amazonense.gapfilled_200_mauve.fasta_2445
VFG007481(gi:27365279)	(fliE) flagellar hook-basal body protein [Flagella (CVF281)] [Vibrio vulnificus CMCP6]	c_amazonense.gapfilled_200_mauve.fasta_2568
VFG007486(gi:28899025)	(flrC) FlaM [Flagella (CVF281)] [Vibrio parahaemolyticus RIMD 2210633]	c_amazonense.gapfilled_200_mauve.fasta_2569
VFG007547(gi:27365304)	(cheW) Chemotaxis signal transduction protein [Flagella (CVF281)] [Vibrio vulnificus CMCP6]	c_amazonense.gapfilled_200_mauve.fasta_897
VFG007662(gi:37678486)	(rmlD) dTDP-4-dehydrorhamnose reductase [Capsular polysaccharide (CVF282)] [Vibrio vulnificus YJ016]	c_amazonense.gapfilled_200_mauve.fasta_3520
VFG007663(gi:37678487)	(rmlC) dTDP-6-deoxy-D-xylo-4-hexulose-3,5-epimerase [Capsular polysaccharide (CVF282)] [Vibrio vulnificus YJ016]	c_amazonense.gapfilled_200_mauve.fasta_3522
VFG007914(gi:120404101)	(ddrA) daunorubicin resistance ABC transporter ATPase subunit [PDIM (phthiocerol dimycocerosate) and PGL (phenolic glycolipid) biosynthesis and transport (CVF288)] [Mycobacterium vanbaalenii PYR-1]	c_amazonense.gapfilled_200_mauve.fasta_2009
VFG007914(gi:120404101)	(ddrA) daunorubicin resistance ABC transporter ATPase subunit [PDIM (phthiocerol dimycocerosate) and PGL (phenolic glycolipid) biosynthesis and transport (CVF288)] [Mycobacterium vanbaalenii PYR-1]	c_amazonense.gapfilled_200_mauve.fasta_2903
VFG008680(gi:433650447)	(ddrA) daunorubicin resistance ABC transporter ATP-binding subunit [PDIM (phthiocerol dimycocerosate) and PGL (phenolic glycolipid) biosynthesis and transport (CVF288)] [Mycobacterium smegmatis JS623	c_amazonense.gapfilled_200_mauve.fasta_1337
VFG008788(gi:433628059)	(mas) Mycocerosic acid synthase [PDIM (phthiocerol dimycocerosate) and PGL (phenolic glycolipid) biosynthesis and transport (CVF288)] [Mycobacterium canettii CIPT 140060008]	c_amazonense.gapfilled_200_mauve.fasta_127
VFG009135(gi:383308045)	(kasB) 3-oxoacyl-(acyl carrier protein) synthase II [FAS-II (CVF300)] [Mycobacterium tuberculosis RGTB327]	c_amazonense.gapfilled_200_mauve.fasta_2971
VFG009305(gi:119870876)	(panC) pantoate--beta-alanine ligase [Pantothenate synthesis (CVF305)] [Mycobacterium sp. KMS]	c_amazonense.gapfilled_200_mauve.fasta_1431
VFG009331(gi:118471351)	(purC) phosphoribosylaminoimidazole-succinocarboxamide synthase [Purine synthesis (CVF306)] [Mycobacterium smegmatis str. MC2 155]	c_amazonense.gapfilled_200_mauve.fasta_56
VFG009376(gi:118470522)	(leuD) 3-isopropylmalate dehydratase, small subunit [Leucine synthesis (CVF309)] [Mycobacterium smegmatis str. MC2 155]	c_amazonense.gapfilled_200_mauve.fasta_2259
VFG009407(gi:145223515)	(glnA1) glutamine synthetase, type I [Glutamine synthesis (CVF311)] [Mycobacterium gilvum PYR-GCK]	c_amazonense.gapfilled_200_mauve.fasta_3113
VFG009865(gi:120405794)	(mprA) two component transcriptional regulator, winged helix family [MprA/B (CVF333)] [Mycobacterium vanbaalenii PYR-1]	c_amazonense.gapfilled_200_mauve.fasta_130
VFG009865(gi:120405794)	(mprA) two component transcriptional regulator, winged helix family [MprA/B (CVF333)] [Mycobacterium vanbaalenii PYR-1]	c_amazonense.gapfilled_200_mauve.fasta_1391
VFG009921(gi:15842122)	(relA) GTP pyrophosphokinase [(p)ppGpp synthesis and hydrolysis (CVF335)] [Mycobacterium tuberculosis CDC1551]	c_amazonense.gapfilled_200_mauve.fasta_3345
VFG010484(gi:54296712)	(htpB) 60 kDa chaperonin (Protein Cpn60)(groEL protein)(Heat shock protein B). [Hsp60 (CVF347)] [Legionella pneumophila str. Paris]	c_amazonense.gapfilled_200_mauve.fasta_3523
VFG010515(gb|YP_095205)	(lidL) Dot/Icm type IV secretion system effector LidL [Dot/Icm (SS047)] [Legionella pneumophila subsp. pneumophila str. Philadelphia 1]	c_amazonense.gapfilled_200_mauve.fasta_44
VFG010782(gi:52841485)	(virB5) LvhB5 [Lvh T4SSA (SS048)] [Legionella pneumophila subsp. pneumophila str. Philadelphia 1]	c_amazonense.gapfilled_200_mauve.fasta_4264
VFG010904(gi:54293730)	(iraB) di/tripeptide transporter homolog IraB [Iron acquisition/assimilation locus (CVF361)] [Legionella pneumophila str. Lens]	c_amazonense.gapfilled_200_mauve.fasta_1083
VFG010904(gi:54293730)	(iraB) di/tripeptide transporter homolog IraB [Iron acquisition/assimilation locus (CVF361)] [Legionella pneumophila str. Lens]	c_amazonense.gapfilled_200_mauve.fasta_384
VFG011154(gi:115421194)	(bplG) probable sugar transferase [LPS (CVF380)] [Bordetella avium 197N]	c_amazonense.gapfilled_200_mauve.fasta_3611
VFG011157(gi:33599142)	(bplF) lipopolysaccharide biosynthesis protein [LPS (CVF380)] [Bordetella bronchiseptica RB50]	c_amazonense.gapfilled_200_mauve.fasta_3612
VFG011157(gi:33599142)	(bplF) lipopolysaccharide biosynthesis protein [LPS (CVF380)] [Bordetella bronchiseptica RB50]	c_amazonense.gapfilled_200_mauve.fasta_606
VFG011167(gi:33594872)	(bplC) lipopolysaccharide biosynthesis protein [LPS (CVF380)] [Bordetella parapertussis 12822]	c_amazonense.gapfilled_200_mauve.fasta_3618
VFG011175(gi:33594874)	(bplA) probable oxidoreductase [LPS (CVF380)] [Bordetella parapertussis 12822]	c_amazonense.gapfilled_200_mauve.fasta_3621
VFG011255(gi:33601532)	(flhB) flagellar biosynthetic protein FlhB [Flagella (CVF382)] [Bordetella bronchiseptica RB50]	c_amazonense.gapfilled_200_mauve.fasta_917
VFG011274(gi:33596137)	(flgB) flagellar basal body rod protein [Flagella (CVF382)] [Bordetella parapertussis 12822]	c_amazonense.gapfilled_200_mauve.fasta_2361
VFG011317(gi:33592490)	(fliR) flagellar biosynthetic protein FliR [Flagella (CVF382)] [Bordetella pertussis Tohama I]	c_amazonense.gapfilled_200_mauve.fasta_2554

VFG011351(gi:33601561)	(fliJ) flagellar protein FliJ [Flagella (CVF382)] [Bordetella bronchiseptica RB50]	c_amazonense.gapfilled_200_mauve.fasta_2563
VFG011355(gi:33601562)	(fliI) flagellum-specific ATP synthase FliI [Flagella (CVF382)] [Bordetella bronchiseptica RB50]	c_amazonense.gapfilled_200_mauve.fasta_2564
VFG011402(gi:23502030)	(fabZ) (3R)-hydroxymyristoyl ACP dehydratase [LPS (CVF383)] [Brucella suis 1330]	c_amazonense.gapfilled_200_mauve.fasta_1592
VFG011430(gb|NP_540392)	(acpXL) acyl carrier protein [LPS (CVF383)] [Brucella melitensis bv. 1 str. 16M]	c_amazonense.gapfilled_200_mauve.fasta_2972
VFG011582(gi:83269014)	(virB9) Type IV secretion system CagX conjugation protein [Type IV secretion system (CVF385)] [Brucella melitensis biovar Abortus 2308]	c_amazonense.gapfilled_200_mauve.fasta_4206
VFG011773(gi:118475094)	(CFF8240_1403) putative heptosyltransferase III waaq [LOS (CVF396)] [Campylobacter fetus subsp. fetus 82-40]	c_amazonense.gapfilled_200_mauve.fasta_667
VFG011805(gi:118475485)	(CFF8240_1412) aspartate racemase [LOS (CVF396)] [Campylobacter fetus subsp. fetus 82-40]	c_amazonense.gapfilled_200_mauve.fasta_102
VFG011809(gi:118475685)	(CFF8240_1413) bifunctional protein HldE [LOS (CVF396)] [Campylobacter fetus subsp. fetus 82-40]	c_amazonense.gapfilled_200_mauve.fasta_368
VFG012080(gi:118475230)	(pglJ) glycosyl transferase, group 1 family protein [N-linked protein glycosylation (CVF400)] [Campylobacter fetus subsp. fetus 82-40]	c_amazonense.gapfilled_200_mauve.fasta_666
VFG012165(gi:110802269)	(CPR_0433) transport protein ysiA [Hemolysin (CVF417)] [Clostridium perfringens SM101]	c_amazonense.gapfilled_200_mauve.fasta_3662
VFG012183(gi:150016581)	(Cbei_1707) hemolysin A [Hemolysin (CVF417)] [Clostridium beijerinckii NCIMB 8052]	c_amazonense.gapfilled_200_mauve.fasta_3796
VFG012187(gi:28210321)	(CTC00586) hemolysin III [Hemolysin (CVF417)] [Clostridium tetani E88]	c_amazonense.gapfilled_200_mauve.fasta_2914
VFG012633(gi:26250982)	(ireA) Putative iron-regulated outer membrane virulence protein [Iron-regulated element (CVF461)] [Escherichia coli CFT073]	c_amazonense.gapfilled_200_mauve.fasta_1319
VFG012837(gi:82524546)	(ipaH2.5) invasion plasmid antigen, fragment [Mxi-Spa TTSS effectors controlled by MxiE (CVF465)] [Shigella dysenteriae Sd197]	c_amazonense.gapfilled_200_mauve.fasta_4033
VFG012837(gi:82524546)	(ipaH2.5) invasion plasmid antigen, fragment [Mxi-Spa TTSS effectors controlled by MxiE (CVF465)] [Shigella dysenteriae Sd197]	c_amazonense.gapfilled_200_mauve.fasta_4046
VFG012837(gi:82524546)	(ipaH2.5) invasion plasmid antigen, fragment [Mxi-Spa TTSS effectors controlled by MxiE (CVF465)] [Shigella dysenteriae Sd197]	c_amazonense.gapfilled_200_mauve.fasta_4118
VFG012837(gi:82524546)	(ipaH2.5) invasion plasmid antigen, fragment [Mxi-Spa TTSS effectors controlled by MxiE (CVF465)] [Shigella dysenteriae Sd197]	c_amazonense.gapfilled_200_mauve.fasta_4122
VFG012837(gi:82524546)	(ipaH2.5) invasion plasmid antigen, fragment [Mxi-Spa TTSS effectors controlled by MxiE (CVF465)] [Shigella dysenteriae Sd197]	c_amazonense.gapfilled_200_mauve.fasta_4144
VFG012874(gi:30064027)	(ipaH) hypothetical protein [Mxi-Spa TTSS effectors controlled by MxiE (CVF465)] [Shigella flexneri 2a str. 2457T]	c_amazonense.gapfilled_200_mauve.fasta_110
VFG013082(gi:82524781)	(virK) VirK [VirK (CVF483)] [Shigella boydii Sb227]	c_amazonense.gapfilled_200_mauve.fasta_757
VFG013147(gi:170719185)	(kdsB) 3-deoxy-D-manno-octulosonate cytidylyltransferase [LOS (CVF494)] [Haemophilus somnus 2336]	c_amazonense.gapfilled_200_mauve.fasta_2916
VFG013165(gi:170717703)	(lpxD) UDP-3-O-(3-hydroxymyristoyl) glucosamine N-acyltransferase [LOS (CVF494)] [Haemophilus somnus 2336]	c_amazonense.gapfilled_200_mauve.fasta_1591
VFG013173(gi:170719107)	(rfaE) rfaE bifunctional protein [LOS (CVF494)] [Haemophilus somnus 2336]	c_amazonense.gapfilled_200_mauve.fasta_2476
VFG013190(gi:170717479)	(hitA) extracellular solute-binding protein family 1 [Haemophilus iron transport locus (CVF501)] [Haemophilus somnus 2336]	c_amazonense.gapfilled_200_mauve.fasta_2334
VFG013191(gi:170717477)	(hitB) binding-protein-dependent transport systems inner membrane component [Haemophilus iron transport locus (CVF501)] [Haemophilus somnus 2336]	c_amazonense.gapfilled_200_mauve.fasta_2333
VFG013192(gi:170717476)	(hitC) ABC transporter related [Haemophilus iron transport locus (CVF501)] [Haemophilus somnus 2336]	c_amazonense.gapfilled_200_mauve.fasta_1027
VFG013192(gi:170717476)	(hitC) ABC transporter related [Haemophilus iron transport locus (CVF501)] [Haemophilus somnus 2336]	c_amazonense.gapfilled_200_mauve.fasta_1338
VFG013192(gi:170717476)	(hitC) ABC transporter related [Haemophilus iron transport locus (CVF501)] [Haemophilus somnus 2336]	c_amazonense.gapfilled_200_mauve.fasta_2332
VFG013192(gi:170717476)	(hitC) ABC transporter related [Haemophilus iron transport locus (CVF501)] [Haemophilus somnus 2336]	c_amazonense.gapfilled_200_mauve.fasta_2580
VFG013192(gi:170717476)	(hitC) ABC transporter related [Haemophilus iron transport locus (CVF501)] [Haemophilus somnus 2336]	c_amazonense.gapfilled_200_mauve.fasta_2707
VFG013192(gi:170717476)	(hitC) ABC transporter related [Haemophilus iron transport locus (CVF501)] [Haemophilus somnus 2336]	c_amazonense.gapfilled_200_mauve.fasta_2717
VFG013192(gi:170717476)	(hitC) ABC transporter related [Haemophilus iron transport locus (CVF501)] [Haemophilus somnus 2336]	c_amazonense.gapfilled_200_mauve.fasta_3314
VFG013197(gi:170717931)	(hemB) Porphobilinogen synthase [Heme biosynthesis (CVF506)] [Haemophilus somnus 2336]	c_amazonense.gapfilled_200_mauve.fasta_1444
VFG013198(gi:170718229)	(hemC) porphobilinogen deaminase [Heme biosynthesis (CVF506)] [Haemophilus somnus 2336]	c_amazonense.gapfilled_200_mauve.fasta_34
VFG013199(gi:170718230)	(hemD) Uroporphyrinogen III synthase HEM4 [Heme biosynthesis (CVF506)] [Haemophilus somnus 2336]	c_amazonense.gapfilled_200_mauve.fasta_35
VFG013203(gi:170719024)	(hemL) glutamate-1-semialdehyde-2,1-aminomutase [Heme biosynthesis (CVF506)] [Haemophilus somnus 2336]	c_amazonense.gapfilled_200_mauve.fasta_2288
VFG013205(gi:170718942)	(hemN) oxygen-independent coproporphyrinogen III oxidase [Heme biosynthesis (CVF506)] [Haemophilus somnus 2336]	c_amazonense.gapfilled_200_mauve.fasta_3232
VFG013247(gi:113460801)	(lpxK) tetraacyldisaccharide 4'-kinase (lipid-A 4'-kinase) [LOS (CVF494)] [Haemophilus somnus 129PT]	c_amazonense.gapfilled_200_mauve.fasta_2918
VFG013252(gi:33152665)	(msbA) ABC transporter ATP-binding protein MsbA [LOS (CVF494)] [Haemophilus ducreyi 35000HP]	c_amazonense.gapfilled_200_mauve.fasta_670
VFG013265(gb|NP_438428)	(orfM) deoxyribonucleotide triphosphate pyrophosphatase [LOS (CVF494)] [Haemophilus influenzae Rd KW20]	c_amazonense.gapfilled_200_mauve.fasta_816
VFG013324(gi:148827923)	(lpxH) UDP-2,3-diacylglucosamine hydrolase [LOS (CVF494)] [Haemophilus influenzae PittGG]	c_amazonense.gapfilled_200_mauve.fasta_2602
VFG013384(gb|NP_439218)	(lpxB) lipid-A-disaccharide synthase [LOS (CVF494)] [Haemophilus influenzae Rd KW20]	c_amazonense.gapfilled_200_mauve.fasta_1594
VFG013394(gi:113461502)	(lpxA) UDP-N-acetylglucosamine acyltransferase [LOS (CVF494)] [Haemophilus somnus 129PT]	c_amazonense.gapfilled_200_mauve.fasta_1593
VFG013407(gi:68249665)	(rfaD) ADP-L-glycero-D-manno-heptose-6-epimerase [LOS (CVF494)] [Haemophilus influenzae 86-028NP]	c_amazonense.gapfilled_200_mauve.fasta_2474
VFG013417(gi:113460510)	(lpxC) UDP-3-O-[3-hydroxymyristoyl] N-acetylglucosamine deacetylase [LOS (CVF494)] [Haemophilus somnus 129PT]	c_amazonense.gapfilled_200_mauve.fasta_3836
VFG013423(gi:113461381)	(gmhA/lpcA) phosphoheptose isomerase [LOS (CVF494)] [Haemophilus somnus 129PT]	c_amazonense.gapfilled_200_mauve.fasta_237
VFG013466(gi:68249928)	(kdsA) 2-dehydro-3-deoxyphosphooctonate aldolase [LOS (CVF494)] [Haemophilus influenzae 86-028NP]	c_amazonense.gapfilled_200_mauve.fasta_3009
VFG013470(gi:113461089)	(kdsA) 2-dehydro-3-deoxyphosphooctonate aldolase [LOS (CVF494)] [Haemophilus somnus 129PT]	c_amazonense.gapfilled_200_mauve.fasta_2141
VFG013515(gi:148826007)	(mrsA/glmM) predicted phosphomannomutase [Exopolysaccharide (CVF495)] [Haemophilus influenzae PittEE]	c_amazonense.gapfilled_200_mauve.fasta_3371
VFG013530(gi:16273471)	(pgi) glucose-6-phosphate isomerase [Exopolysaccharide (CVF495)] [Haemophilus influenzae Rd KW20]	c_amazonense.gapfilled_200_mauve.fasta_18
VFG013535(gi:113461081)	(pgi) glucose-6-phosphate isomerase [Exopolysaccharide (CVF495)] [Haemophilus somnus 129PT]	c_amazonense.gapfilled_200_mauve.fasta_1986
VFG013539(gi:113460750)	(manA) mannose-6-phosphate isomerase (phosphomannose isomerase) [Exopolysaccharide (CVF495)] [Haemophilus somnus 129PT]	c_amazonense.gapfilled_200_mauve.fasta_1104
VFG013568(gi:68248709)	(hitB) iron(III)-transport system permease protein hFbpB [Haemophilus iron transport locus (CVF501)] [Haemophilus influenzae 86-028NP]	c_amazonense.gapfilled_200_mauve.fasta_2693
VFG013573(gi:113460924)	(hitC) iron(III) ABC transporter, ATP-binding protein [Haemophilus iron transport locus (CVF501)] [Haemophilus somnus 129PT]	c_amazonense.gapfilled_200_mauve.fasta_1213
VFG013573(gi:113460924)	(hitC) iron(III) ABC transporter, ATP-binding protein [Haemophilus iron transport locus (CVF501)] [Haemophilus somnus 129PT]	c_amazonense.gapfilled_200_mauve.fasta_2459
VFG013607(gi:113460953)	(hemA) glutamyl-tRNA reductase [Heme biosynthesis (CVF506)] [Haemophilus somnus 129PT]	c_amazonense.gapfilled_200_mauve.fasta_3919
VFG013612(gi:113461676)	(hemE) uroporphyrinogen decarboxylase [Heme biosynthesis (CVF506)] [Haemophilus somnus 129PT]	c_amazonense.gapfilled_200_mauve.fasta_1048
VFG013615(gi:68249709)	(hemH) ferrochelatase [Heme biosynthesis (CVF506)] [Haemophilus influenzae 86-028NP]	c_amazonense.gapfilled_200_mauve.fasta_1950
VFG013618(gi:113461372)	(hemL) glutamate-1-semialdehyde aminotransferase [Heme biosynthesis (CVF506)] [Haemophilus somnus 129PT]	c_amazonense.gapfilled_200_mauve.fasta_28
VFG013629(gi:33152698)	(hemN) coproporphyrinogen III oxidase [Heme biosynthesis (CVF506)] [Haemophilus ducreyi 35000HP]	c_amazonense.gapfilled_200_mauve.fasta_817
VFG013631(gi:30995388)	(hemX) heme biosythesis-like protein [Heme biosynthesis (CVF506)] [Haemophilus influenzae Rd KW20]	c_amazonense.gapfilled_200_mauve.fasta_36
VFG013794(gi:146281442)	(pilC) type 4 pilus biogenesis protein PilC [Type IV pili biosynthesis (CVF518)] [Pseudomonas stutzeri A1501]	c_amazonense.gapfilled_200_mauve.fasta_3404
VFG013800(gi:71735543)	(pilD) type IV pilus prepilin peptidase PilD [Type IV pili biosynthesis (CVF518)] [Pseudomonas syringae pv. phaseolicola 1448A]	c_amazonense.gapfilled_200_mauve.fasta_3403
VFG013811(gi:28868044)	(pilE) type IV pilus biogenesis protein [Type IV pili biosynthesis (CVF518)] [Pseudomonas syringae pv. tomato str. DC3000]	c_amazonense.gapfilled_200_mauve.fasta_3576
VFG013820(gi:146281354)	(pilE) type 4 fimbrial biogenesis protein PilE [Type IV pili biosynthesis (CVF518)] [Pseudomonas stutzeri A1501]	c_amazonense.gapfilled_200_mauve.fasta_2546
VFG013848(gi:152985916)	(pilN) type 4 fimbrial biogenesis protein PilN [Type IV pili biosynthesis (CVF518)] [Pseudomonas aeruginosa PA7]	c_amazonense.gapfilled_200_mauve.fasta_677
VFG013867(gi:77456635)	(pilO) Pilus assembly protein, PilO [Type IV pili biosynthesis (CVF518)] [Pseudomonas fluorescens Pf0-1]	c_amazonense.gapfilled_200_mauve.fasta_676
VFG013881(gi:146305582)	(pilP) Pilus assembly protein, PilQ [Type IV pili biosynthesis (CVF518)] [Pseudomonas mendocina ymp]	c_amazonense.gapfilled_200_mauve.fasta_675
VFG013887(gi:66043674)	(pilQ) type II and III secretion system protein:NolW-like:NolW-like [Type IV pili biosynthesis (CVF518)] [Pseudomonas syringae pv. syringae B728a]	c_amazonense.gapfilled_200_mauve.fasta_674
VFG013907(gi:146309163)	(pilT) twitching motility protein [Type IV pili biosynthesis (CVF518)] [Pseudomonas mendocina ymp]	c_amazonense.gapfilled_200_mauve.fasta_74
VFG013912(gi:28870209)	(pilU) twitching motility protein [Type IV pili biosynthesis (CVF518)] [Pseudomonas syringae pv. tomato str. DC3000]	c_amazonense.gapfilled_200_mauve.fasta_1284
VFG013954(gi:146306666)	(pilZ) type IV pilus assembly PilZ [Type IV pili biosynthesis (CVF518)] [Pseudomonas mendocina ymp]	c_amazonense.gapfilled_200_mauve.fasta_3301
VFG014002(gi:146283959)	(pilS) two-component sensor PilS [Type IV pili biosynthesis (CVF518)] [Pseudomonas stutzeri A1501]	c_amazonense.gapfilled_200_mauve.fasta_3923
VFG014099(gi:15598355)	(wbpA) probable UDP-glucose/GDP-mannose dehydrogenase WbpA [LPS O-antigen (P. aeruginosa) (CVF520)] [Pseudomonas aeruginosa PAO1]	c_amazonense.gapfilled_200_mauve.fasta_3622
VFG014099(gi:15598355)	(wbpA) probable UDP-glucose/GDP-mannose dehydrogenase WbpA [LPS O-antigen (P. aeruginosa) (CVF520)] [Pseudomonas aeruginosa PAO1]	c_amazonense.gapfilled_200_mauve.fasta_435
VFG014106(gi:15598348)	(hisH2) glutamine amidotransferase [LPS O-antigen (P. aeruginosa) (CVF520)] [Pseudomonas aeruginosa PAO1]	c_amazonense.gapfilled_200_mauve.fasta_503
VFG014109(gi:15598345)	(wbpH) probable glycosyltransferase WbpH [LPS O-antigen (P. aeruginosa) (CVF520)] [Pseudomonas aeruginosa PAO1]	c_amazonense.gapfilled_200_mauve.fasta_3614
VFG014116(gi:15598338)	(PA3142) hypothetical protein [LPS O-antigen (P. aeruginosa) (CVF520)] [Pseudomonas aeruginosa PAO1]	c_amazonense.gapfilled_200_mauve.fasta_4148
VFG014147(gi:116051137)	(wbpM) nucleotide sugar epimerase/dehydratase WbpM [LPS O-antigen (P. aeruginosa) (CVF520)] [Pseudomonas aeruginosa UCBPP-PA14]	c_amazonense.gapfilled_200_mauve.fasta_3610
VFG014292(gi:152985351)	(fliC) A-type flagellin [Flagella (CVF521)] [Pseudomonas aeruginosa PA7]	c_amazonense.gapfilled_200_mauve.fasta_3437
VFG014329(gi:146282226)	(fliD) flagellar capping protein FliD [Flagella (CVF521)] [Pseudomonas stutzeri A1501]	c_amazonense.gapfilled_200_mauve.fasta_3434
VFG014378(gi:70729027)	(fleS) sensory box histidine kinase FleS [Flagella (CVF521)] [Pseudomonas fluorescens Pf-5]	c_amazonense.gapfilled_200_mauve.fasta_2996
VFG014561(gi:77457777)	(fliQ) flagellar biosynthesis protein [Flagella (CVF521)] [Pseudomonas fluorescens Pf0-1]	c_amazonense.gapfilled_200_mauve.fasta_2555
VFG014628(gi:146282909)	(fleN) flagellar number regulator FleN [Flagella (CVF521)] [Pseudomonas stutzeri A1501]	c_amazonense.gapfilled_200_mauve.fasta_914
VFG014707(gb|NP_250151)	(motC) flagellar motor protein [Deoxyhexose linking sugar, 209 Da capping structure (AI138)] [Pseudomonas aeruginosa PAO1]	c_amazonense.gapfilled_200_mauve.fasta_912
VFG014731(gi:146307821)	(motD) OmpA/MotB domain protein [Flagella (CVF521)] [Pseudomonas mendocina ymp]	c_amazonense.gapfilled_200_mauve.fasta_2726
VFG014894(gi:152985001)	(algC) phosphomannomutase AlgC [Alginate biosynthesis (CVF522)] [Pseudomonas aeruginosa PA7]	c_amazonense.gapfilled_200_mauve.fasta_2218
VFG014942(gi:77457591)	(mucB) Sigma-E regulatory protein, MucB/RseB [Alginate regulation (CVF523)] [Pseudomonas fluorescens Pf0-1]	c_amazonense.gapfilled_200_mauve.fasta_2170
VFG014950(gb|NP_249457)	(mucD) serine protease MucD precursor [Alginate regulation (CVF523)] [Pseudomonas aeruginosa PAO1]	c_amazonense.gapfilled_200_mauve.fasta_2169
VFG014952(gi:116048682)	(mucD) serine protease MucD precursor [Alginate regulation (CVF523)] [Pseudomonas aeruginosa UCBPP-PA14]	c_amazonense.gapfilled_200_mauve.fasta_2871
VFG014966(gi:28867367)	(algR) alginate biosynthesis regulatory protein AlgR [Alginate regulation (CVF523)] [Pseudomonas syringae pv. tomato str. DC3000]	c_amazonense.gapfilled_200_mauve.fasta_1458
VFG014966(gi:28867367)	(algR) alginate biosynthesis regulatory protein AlgR [Alginate regulation (CVF523)] [Pseudomonas syringae pv. tomato str. DC3000]	c_amazonense.gapfilled_200_mauve.fasta_2756
VFG014966(gi:28867367)	(algR) alginate biosynthesis regulatory protein AlgR [Alginate regulation (CVF523)] [Pseudomonas syringae pv. tomato str. DC3000]	c_amazonense.gapfilled_200_mauve.fasta_2789
VFG014974(gi:146305320)	(algR) two component transcriptional regulator, LytTR family [Alginate regulation (CVF523)] [Pseudomonas mendocina ymp]	c_amazonense.gapfilled_200_mauve.fasta_121
VFG014981(gi:71736095)	(algZ) sensor histidine kinase FimS [Alginate regulation (CVF523)] [Pseudomonas syringae pv. phaseolicola 1448A]	c_amazonense.gapfilled_200_mauve.fasta_3897
VFG015057(gi:152986918)	(lasI) autoinducer synthesis protein LasI [N-(3-oxo-dodecanoyl)-L-homoserine lactone QS system (CVF526)] [Pseudomonas aeruginosa PA7]	c_amazonense.gapfilled_200_mauve.fasta_257
VFG015072(gi:71734710)	(gacS) response regulator, sensor histidine kinase component GacS [GacS/GacA two-component system (CVF529)] [Pseudomonas syringae pv. phaseolicola 1448A]	c_amazonense.gapfilled_200_mauve.fasta_1377
VFG015085(gi:71738055)	(gacA) response regulator, DNA binding component GacA [GacS/GacA two-component system (CVF529)] [Pseudomonas syringae pv. phaseolicola 1448A]	c_amazonense.gapfilled_200_mauve.fasta_2106
VFG015456(gi:71735435)	(hcp1) SciM protein [Hcp secretion island-1 encoded type VI secretion system (H-T6SS) (CVF535)] [Pseudomonas syringae pv. phaseolicola 1448A]	c_amazonense.gapfilled_200_mauve.fasta_781
VFG015475(gi:66048180)	(Psyr_4956) protein of unknown function DUF879 [Hcp secretion island-1 encoded type VI secretion system (H-T6SS) (CVF535)] [Pseudomonas syringae pv. syringae B728a]	c_amazonense.gapfilled_200_mauve.fasta_785
VFG015519(gi:152986215)	(phzF1) probable phenazine biosynthesis protein [Phenazines biosynthesis (CVF536)] [Pseudomonas aeruginosa PA7]	c_amazonense.gapfilled_200_mauve.fasta_820
VFG015521(gb|NP_252906)	(phzG1) phenazine biosynthesis protein PhzG, pyridoxamine 5'-phosphate oxidase [Phenazines biosynthesis (CVF536)] [Pseudomonas aeruginosa PAO1]	c_amazonense.gapfilled_200_mauve.fasta_30
VFG015558(gi:116051721)	(lasB) elastase LasB [Elastase (CVF537)] [Pseudomonas aeruginosa UCBPP-PA14]	c_amazonense.gapfilled_200_mauve.fasta_31
VFG015600(gi:152984031)	(hdtS) probable acyltransferase [Acylhomoserine lactone synthase (CVF528)] [Pseudomonas aeruginosa PA7]	c_amazonense.gapfilled_200_mauve.fasta_1453
VFG015650(gi:167034944)	(flgE) flagellar basal body FlaE domain protein [Flagella (CVF521)] [Pseudomonas putida GB-1]	c_amazonense.gapfilled_200_mauve.fasta_2358
VFG015681(gi:170722859)	(fleR) two component, sigma54 specific, transcriptional regulator, Fis family [Flagella (CVF521)] [Pseudomonas putida W619]	c_amazonense.gapfilled_200_mauve.fasta_1118
VFG015774(gi:167032166)	(mucP) membrane-associated zinc metalloprotease [Alginate regulation (CVF523)] [Pseudomonas putida GB-1]	c_amazonense.gapfilled_200_mauve.fasta_1588
VFG015806(gi:170721686)	(clpV1) type VI secretion ATPase, ClpV1 family [Hcp secretion island-1 encoded type VI secretion system (H-T6SS) (CVF535)] [Pseudomonas putida W619]	c_amazonense.gapfilled_200_mauve.fasta_788
VFG015881(gi:28871838)	(PSPTO_4713) alanyl tRNA synthetase-related protein [Phytotoxin coronatine (CVF545)] [Pseudomonas syringae pv. tomato str. DC3000]	c_amazonense.gapfilled_200_mauve.fasta_215
VFG015881(gi:28871838)	(PSPTO_4713) alanyl tRNA synthetase-related protein [Phytotoxin coronatine (CVF545)] [Pseudomonas syringae pv. tomato str. DC3000]	c_amazonense.gapfilled_200_mauve.fasta_3331
VFG015885(gi:71736839)	(cysC1) adenylylsulfate kinase [Phytotoxin phaseolotoxin (CVF546)] [Pseudomonas syringae pv. phaseolicola 1448A]	c_amazonense.gapfilled_200_mauve.fasta_2222
VFG015888(gi:71737277)	(PSPPH_4304) hydrolase, HAD-superfamily, subfamily IIA [Phytotoxin phaseolotoxin (CVF546)] [Pseudomonas syringae pv. phaseolicola 1448A]	c_amazonense.gapfilled_200_mauve.fasta_2838
VFG015903(gi:71735587)	(argK) phaseolotoxin-insensitive ornithine carbamoyltransferase [Phytotoxin phaseolotoxin (CVF546)] [Pseudomonas syringae pv. phaseolicola 1448A]	c_amazonense.gapfilled_200_mauve.fasta_2187
VFG015903(gi:71735587)	(argK) phaseolotoxin-insensitive ornithine carbamoyltransferase [Phytotoxin phaseolotoxin (CVF546)] [Pseudomonas syringae pv. phaseolicola 1448A]	c_amazonense.gapfilled_200_mauve.fasta_248
VFG015903(gi:71735587)	(argK) phaseolotoxin-insensitive ornithine carbamoyltransferase [Phytotoxin phaseolotoxin (CVF546)] [Pseudomonas syringae pv. phaseolicola 1448A]	c_amazonense.gapfilled_200_mauve.fasta_3357
VFG015914(gi:15597389)	(hcnA) hydrogen cyanide synthase HcnA [Hydrogen cyanide production (CVF549)] [Pseudomonas aeruginosa PAO1]	c_amazonense.gapfilled_200_mauve.fasta_2829
VFG015925(gi:77459737)	(hcnB) BFD-like (2Fe-2S)-binding region [Hydrogen cyanide production (CVF549)] [Pseudomonas fluorescens Pf0-1]	c_amazonense.gapfilled_200_mauve.fasta_2830
VFG015927(gi:152985611)	(hcnC) hydrogen cyanide synthase HcnC [Hydrogen cyanide production (CVF549)] [Pseudomonas aeruginosa PA7]	c_amazonense.gapfilled_200_mauve.fasta_2328
VFG015930(gi:70729947)	(hcnC) hydrogen cyanide synthase HcnC [Hydrogen cyanide production (CVF549)] [Pseudomonas fluorescens Pf-5]	c_amazonense.gapfilled_200_mauve.fasta_2831
VFG015972(gi:146307893)	(pvdA) Lysine/ornithine N-monooxygenase-like protein [Pyoverdine (CVF551)] [Pseudomonas mendocina ymp]	c_amazonense.gapfilled_200_mauve.fasta_562
VFG016039(gi:152983967)	(pvdJ) thermophilic carboxylesterase Est2 [Pyoverdine (CVF551)] [Pseudomonas aeruginosa PA7]	c_amazonense.gapfilled_200_mauve.fasta_238
VFG016057(gi:146307890)	(pvdH) 2,4-diaminobutyrate 4-transaminase [Pyoverdine (CVF551)] [Pseudomonas mendocina ymp]	c_amazonense.gapfilled_200_mauve.fasta_563

VFG016303(gi:16078902)	(capD) gamma-glutamyltranspeptidase [Polyglutamic acid capsule (CVF566)] [Bacillus subtilis subsp. subtilis str. 168]	c_amazonense.gapfilled_200_mauve.fasta_1259
VFG016387(gi:42784426)	(galE) UDP-glucose 4-epimerase [Polysaccharide capsule (CVF567)] [Bacillus cereus ATCC 10987]	c_amazonense.gapfilled_200_mauve.fasta_425
VFG016390(gi:42784429)	(BCE_5384) NAD dependent epimerase/dehydratase family protein [Polysaccharide capsule (CVF567)] [Bacillus cereus ATCC 10987]	c_amazonense.gapfilled_200_mauve.fasta_664
VFG016412(gi:49478940)	(BT9727_4951) conserved hypothetical protein, possible MVIN-like virulence factor [Polysaccharide capsule (CVF567)] [Bacillus thuringiensis serovar konkukian str. 97-27]	c_amazonense.gapfilled_200_mauve.fasta_3327
VFG016433(gi:118480317)	(gtaB) UTP--glucose-1-phosphate uridylyltransferase [Polysaccharide capsule (CVF567)] [Bacillus thuringiensis str. Al Hakam]	c_amazonense.gapfilled_200_mauve.fasta_3461
VFG016433(gi:118480317)	(gtaB) UTP--glucose-1-phosphate uridylyltransferase [Polysaccharide capsule (CVF567)] [Bacillus thuringiensis str. Al Hakam]	c_amazonense.gapfilled_200_mauve.fasta_430
VFG016485(gi:31544430)	(tuf) elongation factor Tu [EF-Tu (CVF587)] [Mycoplasma gallisepticum str. R(low)]	c_amazonense.gapfilled_200_mauve.fasta_3696
VFG016485(gi:31544430)	(tuf) elongation factor Tu [EF-Tu (CVF587)] [Mycoplasma gallisepticum str. R(low)]	c_amazonense.gapfilled_200_mauve.fasta_3708
VFG016486(gi:54020567)	(tuf) elongation factor Tu [EF-Tu (CVF587)] [Mycoplasma hyopneumoniae 232]	c_amazonense.gapfilled_200_mauve.fasta_1780
VFG016676(gi:49475416)	(omp89) Outer membrane protein [Fibronectin-binding protein (AI257)] [Bartonella henselae str. Houston-1]	c_amazonense.gapfilled_200_mauve.fasta_1589
VFG017360(gi:153950020)	(ddhA) glucose-1-phosphate cytidylyltransferase [O-antigen (CVF043)] [Yersinia pseudotuberculosis IP 31758]	c_amazonense.gapfilled_200_mauve.fasta_3452
VFG017486(gi:118462718)	(nuoG) NADH-quinone oxidoreductase, chain g [NuoG (CVF620)] [Mycobacterium avium 104]	c_amazonense.gapfilled_200_mauve.fasta_835
VFG017670(gi:443489219)	(icl) isocitrate lyase Icl [Isocitrate lyase (CVF302)] [Mycobacterium liflandii 128FXT]	c_amazonense.gapfilled_200_mauve.fasta_2173
VFG017733(gi:153952077)	(pebA) amino acid ABC transporter, periplasmic amino acid-binding protein PEB1 [PEB1/CBF1 (CVF388)] [Campylobacter jejuni subsp. doylei 269.97]	c_amazonense.gapfilled_200_mauve.fasta_95
VFG017759(gi:153951764)	(kpsF) arabinose-5-phosphate isomerase [Capsule biosynthesis and transport (CVF393)] [Campylobacter jejuni subsp. doylei 269.97]	c_amazonense.gapfilled_200_mauve.fasta_2899
VFG017761(gi:153952331)	(kpsE) capsular polysaccharide ABC transporter [Capsule biosynthesis and transport (CVF393)] [Campylobacter jejuni subsp. doylei 269.97]	c_amazonense.gapfilled_200_mauve.fasta_437
VFG017855(gi:153948767)	(pilW) putative transposase [Type IV pili (CVF049)] [Yersinia pseudotuberculosis IP 31758]	c_amazonense.gapfilled_200_mauve.fasta_1835
VFG017855(gi:153948767)	(pilW) putative transposase [Type IV pili (CVF049)] [Yersinia pseudotuberculosis IP 31758]	c_amazonense.gapfilled_200_mauve.fasta_3940
VFG017855(gi:153948767)	(pilW) putative transposase [Type IV pili (CVF049)] [Yersinia pseudotuberculosis IP 31758]	c_amazonense.gapfilled_200_mauve.fasta_4305
VFG017989(gi:157415665)	(C8J_1346) hypothetical protein [Capsule biosynthesis and transport (CVF393)] [Campylobacter jejuni subsp. jejuni 81116]	c_amazonense.gapfilled_200_mauve.fasta_3443
VFG017989(gi:157415665)	(C8J_1346) hypothetical protein [Capsule biosynthesis and transport (CVF393)] [Campylobacter jejuni subsp. jejuni 81116]	c_amazonense.gapfilled_200_mauve.fasta_603
VFG018000(gi:157415395)	(C8J_1075) hypothetical protein [LOS (CVF396)] [Campylobacter jejuni subsp. jejuni 81116]	c_amazonense.gapfilled_200_mauve.fasta_137
VFG018020(gi:157415415)	(gmhA-1) phosphoheptose isomerase [LOS (CVF396)] [Campylobacter jejuni subsp. jejuni 81116]	c_amazonense.gapfilled_200_mauve.fasta_531
VFG018293(gi:161620106)	(dhbB) Probable isochorismatase [Brucebactin (CVF386)] [Brucella canis ATCC 23365]	c_amazonense.gapfilled_200_mauve.fasta_1311
VFG018336(gi:161612541)	(staB) hypothetical protein [Sta (CVF019)] [Salmonella enterica subsp. enterica serovar Paratyphi B str. SPB7]	c_amazonense.gapfilled_200_mauve.fasta_3129
VFG018402(gi:161505694)	(mgtB) hypothetical protein [Mg2+ transport (CVF005)] [Salmonella enterica subsp. arizonae serovar 62:z4,z23:-- str. RSK2980]	c_amazonense.gapfilled_200_mauve.fasta_2531
VFG018554(gi:161502085)	(prgK) hypothetical protein [TTSS (SPI-1 encode) (CVF031)] [Salmonella enterica subsp. arizonae serovar 62:z4,z23:-- str. RSK2980]	c_amazonense.gapfilled_200_mauve.fasta_1719
VFG018558(gi:161502087)	(orgB) hypothetical protein [TTSS (SPI-1 encode) (CVF031)] [Salmonella enterica subsp. arizonae serovar 62:z4,z23:-- str. RSK2980]	c_amazonense.gapfilled_200_mauve.fasta_1718
VFG018707(gi:163869212)	(korB) trans-acting regulatory protein [Trw type IV secretion system (CVF604)] [Bartonella tribocorum CIP 105476]	c_amazonense.gapfilled_200_mauve.fasta_1236
VFG018707(gi:163869212)	(korB) trans-acting regulatory protein [Trw type IV secretion system (CVF604)] [Bartonella tribocorum CIP 105476]	c_amazonense.gapfilled_200_mauve.fasta_4139
VFG018707(gi:163869212)	(korB) trans-acting regulatory protein [Trw type IV secretion system (CVF604)] [Bartonella tribocorum CIP 105476]	c_amazonense.gapfilled_200_mauve.fasta_4198
VFG018806(gi:161870575)	(exbD) biopolymer transport protein [Ton system (CVF202)] [Neisseria meningitidis 053442]	c_amazonense.gapfilled_200_mauve.fasta_1497
VFG018806(gi:161870575)	(exbD) biopolymer transport protein [Ton system (CVF202)] [Neisseria meningitidis 053442]	c_amazonense.gapfilled_200_mauve.fasta_1508
VFG018806(gi:161870575)	(exbD) biopolymer transport protein [Ton system (CVF202)] [Neisseria meningitidis 053442]	c_amazonense.gapfilled_200_mauve.fasta_1509
VFG019028(gi:170025406)	(YPK_3189) DegT/DnrJ/EryC1/StrS aminotransferase [O-antigen (CVF043)] [Yersinia pseudotuberculosis YPIII]	c_amazonense.gapfilled_200_mauve.fasta_3450
VFG019252(gi:163844264)	(virB4) type IV secretion/conjugal transfer ATPase, VirB4 family [Type IV secretion system (CVF385)] [Brucella suis ATCC 23445]	c_amazonense.gapfilled_200_mauve.fasta_4222
VFG019276(gi:163844212)	(dhbA) hypothetical protein [Brucebactin (CVF386)] [Brucella suis ATCC 23445]	c_amazonense.gapfilled_200_mauve.fasta_1310
VFG019334(gi:225685884)	(dhbC) isochorismate synthase [Brucebactin (CVF386)] [Brucella melitensis ATCC 23457]	c_amazonense.gapfilled_200_mauve.fasta_1313
VFG019335(gi:225685883)	(dhbE) 2,3-dihydroxybenzoate-AMP ligase [Brucebactin (CVF386)] [Brucella melitensis ATCC 23457]	c_amazonense.gapfilled_200_mauve.fasta_1312
VFG019385(gi:226224673)	(bsh) conjugated bile acid hydrolase [Bile-salt hydrolase (CVF250)] [Listeria monocytogenes serotype 4b str. CLIP 80459]	c_amazonense.gapfilled_200_mauve.fasta_3152
VFG019431(gi:188527050)	(napA) neutrophil activating protein (napA) (bacterioferritin) [Neutrophil-activating protein (HP-NAP) (CVF215)] [Helicobacter pylori Shi470]	c_amazonense.gapfilled_200_mauve.fasta_3755
VFG019544(gi:210134297)	(futC) alpha-1,2-fucosyltransferase [Lipopolysaccharide Lewis antigens (CVF222)] [Helicobacter pylori P12]	c_amazonense.gapfilled_200_mauve.fasta_3528
VFG019722(gi:194099155)	(exbB) ExbB [Ton system (CVF202)] [Neisseria gonorrhoeae NCCP11945]	c_amazonense.gapfilled_200_mauve.fasta_1507
VFG019732(gi:229588336)	(pilD) type IV prepilin-like proteins leader peptide processing enzyme [Type IV pili biosynthesis (CVF518)] [Pseudomonas fluorescens SBW25]	c_amazonense.gapfilled_200_mauve.fasta_1867
VFG020307(gi:186894378)	(YPTS_1052) glycosyl transferase group 1 [O-antigen (CVF043)] [Yersinia pseudotuberculosis PB1/+]	c_amazonense.gapfilled_200_mauve.fasta_3537
VFG020395(gi:197248938)	(prgI) type III secretion apparatus needle protein [TTSS (SPI-1 encode) (CVF031)] [Salmonella enterica subsp. enterica serovar Agona str. SL483]	c_amazonense.gapfilled_200_mauve.fasta_1721
VFG020437(gi:194736333)	(iagB) invasion protein IagB [TTSS (SPI-1 encode) (CVF031)] [Salmonella enterica subsp. enterica serovar Schwarzengrund str. CVM19633]	c_amazonense.gapfilled_200_mauve.fasta_4291
VFG021683(gi:197251878)	(stjC) putative periplasmic chaperone protein [Stj (CVF028)] [Salmonella enterica subsp. enterica serovar Agona str. SL483]	c_amazonense.gapfilled_200_mauve.fasta_1325
VFG021856(gi:118470693)	(fxbA) Formyl transferase [exochelin (IA032)] [Mycobacterium smegmatis str. MC2 155]	c_amazonense.gapfilled_200_mauve.fasta_3767
VFG022587(gi:333989098)	(icl) isocitrate lyase Icl [Isocitrate lyase (CVF302)] [Mycobacterium sp. JDM601]	c_amazonense.gapfilled_200_mauve.fasta_1439
VFG022623(gi:333992530)	(panD) aspartate 1-decarboxylase precursor PanD [Pantothenate synthesis (CVF305)] [Mycobacterium sp. JDM601]	c_amazonense.gapfilled_200_mauve.fasta_1432
VFG022639(gi:333989135)	(proC) pyrroline-5-carboxylate reductase ProC [Proline synthesis (CVF307)] [Mycobacterium sp. JDM601]	c_amazonense.gapfilled_200_mauve.fasta_70
VFG022709(gi:169629208)	(mbtE) putative peptide synthetase MbtE [Mycobactin (CVF315)] [Mycobacterium abscessus ATCC 19977]	c_amazonense.gapfilled_200_mauve.fasta_267
VFG022816(gi:169630604)	(narK2) integral membrane nitrite extrusion protein NarK3 [Nitrate/nitrite transporter (CVF320)] [Mycobacterium abscessus ATCC 19977]	c_amazonense.gapfilled_200_mauve.fasta_3467
VFG023783(gi:332161122)	(YE105_C1500) Mannose-1-phosphate guanylyltransferase, ManC protein [O-antigen (CVF043)] [Yersinia enterocolitica subsp. palearctica 105.5R(r)]	c_amazonense.gapfilled_200_mauve.fasta_3538
VFG023786(gi:332161126)	(YE105_C1504) GDP-mannose 4,6-dehydratase [O-antigen (CVF043)] [Yersinia enterocolitica subsp. palearctica 105.5R(r)]	c_amazonense.gapfilled_200_mauve.fasta_3530
VFG023952(gi:315443890)	(trpD) anthranilate phosphoribosyltransferase [Tryptophan synthesis (CVF308)] [Mycobacterium gilvum Spyr1]	c_amazonense.gapfilled_200_mauve.fasta_2217
VFG024922(gi:170738260)	(pilN) type IVB pilus formation outer membrane protein [Type IV pili (CVF638)] [Burkholderia cenocepacia MC0-3]	c_amazonense.gapfilled_200_mauve.fasta_4217
VFG025389(gi:53723902)	(fliM) flagellar motor switch protein FliM [Flagella (CVF643)] [Burkholderia mallei ATCC 23344]	c_amazonense.gapfilled_200_mauve.fasta_2560
VFG025410(gi:83721171)	(fliN) flagellar motor switch protein FliN [Flagella (CVF643)] [Burkholderia thailandensis E264]	c_amazonense.gapfilled_200_mauve.fasta_2559
VFG025537(gi:284159411)	(fliF) flagellar MS-ring protein [Flagella (CVF643)] [Burkholderia thailandensis E264]	c_amazonense.gapfilled_200_mauve.fasta_2567
VFG025667(gi:83720527)	(flgF) flagellar basal body rod protein FlgF [Flagella (CVF643)] [Burkholderia thailandensis E264]	c_amazonense.gapfilled_200_mauve.fasta_2357
VFG025694(gi:107023949)	(flgI) flagellar basal body P-ring protein [Flagella (CVF643)] [Burkholderia cenocepacia AU 1054]	c_amazonense.gapfilled_200_mauve.fasta_2354
VFG025719(gi:83719486)	(flgJ) flagellar rod assembly protein/muramidase FlgJ [Flagella (CVF643)] [Burkholderia thailandensis E264]	c_amazonense.gapfilled_200_mauve.fasta_2353
VFG025722(gi:206558976)	(flgK) flagellar hook-associated protein FlgK [Flagella (CVF643)] [Burkholderia cenocepacia J2315]	c_amazonense.gapfilled_200_mauve.fasta_2352
VFG025797(gi:83719420)	(flhA) flagellar biosynthesis protein FlhA [Flagella (CVF643)] [Burkholderia thailandensis E264]	c_amazonense.gapfilled_200_mauve.fasta_916
VFG025816(gi:124386218)	(cheZ) chemotaxis regulator CheZ [Flagella (CVF643)] [Burkholderia mallei NCTC 10229]	c_amazonense.gapfilled_200_mauve.fasta_3000
VFG025862(gi:83721660)	(cheD) chemoreceptor glutamine deamidase CheD [Flagella (CVF643)] [Burkholderia thailandensis E264]	c_amazonense.gapfilled_200_mauve.fasta_895
VFG025866(gi:170731596)	(cheR) chemotaxis protein CheR [Flagella (CVF643)] [Burkholderia cenocepacia MC0-3]	c_amazonense.gapfilled_200_mauve.fasta_3276
VFG025879(gi:170731595)	(tsr) methyl-accepting chemotaxis sensory transducer [Flagella (CVF643)] [Burkholderia cenocepacia MC0-3]	c_amazonense.gapfilled_200_mauve.fasta_1812
VFG025917(gi:206558534)	(cheY1) chemotaxis two-component response regulator CheY [Flagella (CVF643)] [Burkholderia cenocepacia J2315]	c_amazonense.gapfilled_200_mauve.fasta_1815
VFG025917(gi:206558534)	(cheY1) chemotaxis two-component response regulator CheY [Flagella (CVF643)] [Burkholderia cenocepacia J2315]	c_amazonense.gapfilled_200_mauve.fasta_901
VFG025942(gi:206558532)	(motA) flagellar motor protein MotA [Flagella (CVF643)] [Burkholderia cenocepacia J2315]	c_amazonense.gapfilled_200_mauve.fasta_1557
VFG026061(gi:107021859)	(wcbT) 8-amino-7-oxononanoate synthase [Capsule I (CVF645)] [Burkholderia cenocepacia AU 1054]	c_amazonense.gapfilled_200_mauve.fasta_3478
VFG026063(gi:206561564)	(wcbT) putative acyl-CoA transferase [Capsule I (CVF645)] [Burkholderia cenocepacia J2315]	c_amazonense.gapfilled_200_mauve.fasta_1446
VFG026063(gi:206561564)	(wcbT) putative acyl-CoA transferase [Capsule I (CVF645)] [Burkholderia cenocepacia J2315]	c_amazonense.gapfilled_200_mauve.fasta_3875
VFG026099(gi:83721152)	(wcbR) type I polyketide synthase WcbR [Capsule I (CVF645)] [Burkholderia thailandensis E264]	c_amazonense.gapfilled_200_mauve.fasta_1572
VFG026414(gi:433649359)	(lysA) diaminopimelate decarboxylase [Lysine synthesis (CVF310)] [Mycobacterium smegmatis JS623]	c_amazonense.gapfilled_200_mauve.fasta_3334
VFG026433(gi:383308019)	(glnA1) glutamine synthetase [Glutamine synthesis (CVF311)] [Mycobacterium tuberculosis RGTB327]	c_amazonense.gapfilled_200_mauve.fasta_1555
VFG026433(gi:383308019)	(glnA1) glutamine synthetase [Glutamine synthesis (CVF311)] [Mycobacterium tuberculosis RGTB327]	c_amazonense.gapfilled_200_mauve.fasta_3794
VFG026463(gi:479055768)	(mgtC) putative Mg2+ TRANSPORT P-TYPE ATPASE C MGTC [Magnesium transport (CVF313)] [Mycobacterium tuberculosis 7199-99]	c_amazonense.gapfilled_200_mauve.fasta_3029
VFG026700(gi:494692156)	(mbtI) anthranilate synthase component I [Mycobactin (CVF315)] [Mycobacterium tuberculosis str. Haarlem/NITR202]	c_amazonense.gapfilled_200_mauve.fasta_1932
VFG029181(gi:494691572)	(fadE14) acyl-CoA dehydrogenase FADE13 [Mycobactin (CVF315)] [Mycobacterium tuberculosis str. Haarlem/NITR202]	c_amazonense.gapfilled_200_mauve.fasta_2239
VFG029717(gi:386005483)	(mps1) fatty-acid-CoA ligase [GPL locus (CVF650)] [Mycobacterium tuberculosis RGTB423]	c_amazonense.gapfilled_200_mauve.fasta_3549
VFG029768(gi:145221013)	(fadE5) acyl-CoA dehydrogenase domain-containing protein [GPL locus (CVF650)] [Mycobacterium gilvum PYR-GCK]	c_amazonense.gapfilled_200_mauve.fasta_3393
VFG029797(gi:397680665)	(fadE5) acyl-CoA dehydrogenase [GPL locus (CVF650)] [Mycobacterium abscessus subsp. bolletii str. GO 06]	c_amazonense.gapfilled_200_mauve.fasta_2744
VFG029832(gi:379753832)	(pks) beta-ketoacyl synthase [GPL locus (CVF650)] [Mycobacterium intracellulare MOTT-02]	c_amazonense.gapfilled_200_mauve.fasta_1425
VFG030286(gi:15610223)	(adhD) Probable zinc-type alcohol dehydrogenase AdhD (aldehyde reductase) [MymA operon (CVF649)] [Mycobacterium tuberculosis H37Rv]	c_amazonense.gapfilled_200_mauve.fasta_2179
VFG030293(gi:108801614)	(adhD) alcohol dehydrogenase GroES-like protein [MymA operon (CVF649)] [Mycobacterium sp. MCS]	c_amazonense.gapfilled_200_mauve.fasta_1447
VFG030387(gi:433645508)	(tgs4) acyl-CoA synthetase (AMP-forming)/AMP-acid ligase II [MymA operon (CVF649)] [Mycobacterium smegmatis JS623]	c_amazonense.gapfilled_200_mauve.fasta_2862
VFG030388(gi:15610226)	(fadD13) Probable chain-fatty-acid-CoA ligase FadD13 (fatty-acyl-CoA synthetase) [MymA operon (CVF649)] [Mycobacterium tuberculosis H37Rv]	c_amazonense.gapfilled_200_mauve.fasta_261
VFG030400(gi:333989012)	(fadD13) fatty-acid--CoA ligase [MymA operon (CVF649)] [Mycobacterium sp. JDM601]	c_amazonense.gapfilled_200_mauve.fasta_1285
VFG030400(gi:333989012)	(fadD13) fatty-acid--CoA ligase [MymA operon (CVF649)] [Mycobacterium sp. JDM601]	c_amazonense.gapfilled_200_mauve.fasta_2749
VFG030400(gi:333989012)	(fadD13) fatty-acid--CoA ligase [MymA operon (CVF649)] [Mycobacterium sp. JDM601]	c_amazonense.gapfilled_200_mauve.fasta_743
VFG030565(gi:31793499)	(sugA) sugar-transport integral membrane protein ABC transporter UspA [Trehalose-recycling ABC transporter (CVF651)] [Mycobacterium bovis AF2122/97]	c_amazonense.gapfilled_200_mauve.fasta_169
VFG030609(gi:383308107)	(sugA) hypothetical protein [Trehalose-recycling ABC transporter (CVF651)] [Mycobacterium tuberculosis RGTB327]	c_amazonense.gapfilled_200_mauve.fasta_2369
VFG030609(gi:383308107)	(sugA) hypothetical protein [Trehalose-recycling ABC transporter (CVF651)] [Mycobacterium tuberculosis RGTB327]	c_amazonense.gapfilled_200_mauve.fasta_3239
VFG030630(gi:120403530)	(sugB) binding-protein-dependent transport system inner membrane protein [Trehalose-recycling ABC transporter (CVF651)] [Mycobacterium vanbaalenii PYR-1]	c_amazonense.gapfilled_200_mauve.fasta_3238
VFG030632(gi:169628465)	(sugB) Probable sugar ABC transporter, permease protein SugB [Trehalose-recycling ABC transporter (CVF651)] [Mycobacterium abscessus ATCC 19977]	c_amazonense.gapfilled_200_mauve.fasta_2641
VFG030658(gi:385990670)	(sugB) sugar-transport integral membrane protein ABC transporter sugB [Trehalose-recycling ABC transporter (CVF651)] [Mycobacterium tuberculosis CCDC5180]	c_amazonense.gapfilled_200_mauve.fasta_2370
VFG030681(gi:119870084)	(sugC) ABC transporter-like protein [Trehalose-recycling ABC transporter (CVF651)] [Mycobacterium sp. KMS]	c_amazonense.gapfilled_200_mauve.fasta_3712
VFG030692(gi:333989849)	(sugC) sugar ABC transporter [Trehalose-recycling ABC transporter (CVF651)] [Mycobacterium sp. JDM601]	c_amazonense.gapfilled_200_mauve.fasta_3237
VFG030694(gi:315444768)	(sugC) sugar ABC transporter ATP-binding protein [Trehalose-recycling ABC transporter (CVF651)] [Mycobacterium gilvum Spyr1]	c_amazonense.gapfilled_200_mauve.fasta_714
VFG030696(gi:387874659)	(sugC) ABC transporter, ATP-binding protein SugC [Trehalose-recycling ABC transporter (CVF651)] [Mycobacterium intracellulare str. MOTT36Y]	c_amazonense.gapfilled_200_mauve.fasta_2794
VFG030697(gi:379753298)	(sugC) ABC transporter, ATP-binding protein SugC [Trehalose-recycling ABC transporter (CVF651)] [Mycobacterium intracellulare MOTT-02]	c_amazonense.gapfilled_200_mauve.fasta_2835
VFG030700(gi:507420313)	(sugC) sugar ABC transporter ATP-binding protein SugC [Trehalose-recycling ABC transporter (CVF651)] [Mycobacterium abscessus subsp. bolletii 50594]	c_amazonense.gapfilled_200_mauve.fasta_166
VFG030700(gi:507420313)	(sugC) sugar ABC transporter ATP-binding protein SugC [Trehalose-recycling ABC transporter (CVF651)] [Mycobacterium abscessus subsp. bolletii 50594]	c_amazonense.gapfilled_200_mauve.fasta_1674
VFG031050(gi:169631610)	(zmp1) Probable zinc metalloprotease [Zn++ metallophrotease (CVF655)] [Mycobacterium abscessus ATCC 19977]	c_amazonense.gapfilled_200_mauve.fasta_1510
VFG031050(gi:169631610)	(zmp1) Probable zinc metalloprotease [Zn++ metallophrotease (CVF655)] [Mycobacterium abscessus ATCC 19977]	c_amazonense.gapfilled_200_mauve.fasta_3800
VFG031350(gi:15827315)	(ctpC) cation transport ATPase [P(1)-type Mn2+ transporting ATPase (CVF657)] [Mycobacterium leprae TN]	c_amazonense.gapfilled_200_mauve.fasta_1075
VFG031473(gi:169628696)	(ndk) Probable nucleoside diphosphate kinase (NDK) [Nucleoside diphosphate kinase (CVF660)] [Mycobacterium abscessus ATCC 19977]	c_amazonense.gapfilled_200_mauve.fasta_1168
VFG031514(gi:41408083)	(ptpA) PtpA [Tyrosine phosphatase (CVF661)] [Mycobacterium avium subsp. paratuberculosis K-10]	c_amazonense.gapfilled_200_mauve.fasta_2571
VFG031685(gi:169631127)	(senX3) Sensor-like histidine kinase senX3 [SenX3 (CVF666)] [Mycobacterium abscessus ATCC 19977]	c_amazonense.gapfilled_200_mauve.fasta_451
VFG031723(gi:15839882)	(regX3) DNA-binding response regulator RegX3 [RegX3 (CVF667)] [Mycobacterium tuberculosis CDC1551]	c_amazonense.gapfilled_200_mauve.fasta_450
VFG031738(gi:169631126)	(regX3) Sensory transduction protein RegX3 [RegX3 (CVF667)] [Mycobacterium abscessus ATCC 19977]	c_amazonense.gapfilled_200_mauve.fasta_1880
VFG031961(gi:289434920)	(lap) bifunctional aldehyde/alcohol dehydrogenase [Listeria adhesion protein (CVF228)] [Listeria seeligeri serovar 1/2b str. SLCC3954]	c_amazonense.gapfilled_200_mauve.fasta_2132
VFG032254(gi:347549588)	(oppA) putative pheromone ABC transporter binding protein [Oligopeptide-binding protein (CVF240)] [Listeria ivanovii subsp. ivanovii PAM 55]	c_amazonense.gapfilled_200_mauve.fasta_3828
VFG032255(gi:289435537)	(oppA) oligopeptide ABC transporter substrate-binding protein [Oligopeptide-binding protein (CVF240)] [Listeria seeligeri serovar 1/2b str. SLCC3954]	c_amazonense.gapfilled_200_mauve.fasta_2986
VFG032386(gi:347549217)	(stp) putative phosphoprotein phosphatase [Serine-threonine phosphatase (CVF245)] [Listeria ivanovii subsp. ivanovii PAM 55]	c_amazonense.gapfilled_200_mauve.fasta_3423
VFG032467(gi:289435743)	(lgt) prolipoprotein diacylglyceryl transferase [Lipoprotein diacylglyceryl transferase (CVF248)] [Listeria seeligeri serovar 1/2b str. SLCC3954]	c_amazonense.gapfilled_200_mauve.fasta_3145
VFG032760(gi:347548107)	(cheY) putative chemotaxis response regulator CheY [CheA/CheY (CVF255)] [Listeria ivanovii subsp. ivanovii PAM 55]	c_amazonense.gapfilled_200_mauve.fasta_1935
VFG033234(gi:410480369)	(fimI) long polar fimbrial protein LpfA [Type I fimbriae (CVF426)] [Escherichia coli O104:H4 str. 2009EL-2050]	c_amazonense.gapfilled_200_mauve.fasta_3121

VFG033234(gi:410480369)	(fimI) long polar fimbrial protein LpfA [Type I fimbriae (CVF426)] [Escherichia coli O104:H4 str. 2009EL-2050]	c_amazonense.gapfilled_200_mauve.fasta_3125
VFG033876(gi:386641995)	(hlyD) hemolysin secretion protein D [Alpha-hemolysin (CVF453)] [Escherichia coli ABU 83972]	c_amazonense.gapfilled_200_mauve.fasta_1661
VFG033882(gi:260718956)	(hlyB) hemolysin B [Alpha-hemolysin (CVF453)] [Escherichia coli O103:H2 str. 12009]	c_amazonense.gapfilled_200_mauve.fasta_1662
VFG033884(gi:386641996)	(hlyB) hemolysin secretion protein HlyB [Alpha-hemolysin (CVF453)] [Escherichia coli ABU 83972]	c_amazonense.gapfilled_200_mauve.fasta_1302
VFG034292(gi:386621154)	(chuT) Periplasmic hemin-binding protein [Hemin uptake (CVF460)] [Escherichia coli NA114]	c_amazonense.gapfilled_200_mauve.fasta_380
VFG034657(gi:215485614)	(ibeB) copper/silver efflux system outer membrane protein CusC [Invasion of brain endothelial cells (Ibes) (CVF429)] [Escherichia coli O127:H6 str. E2348/69]	c_amazonense.gapfilled_200_mauve.fasta_1423
VFG034684(gi:222155327)	(ibeB) Cation efflux system protein cusC [Invasion of brain endothelial cells (Ibes) (CVF429)] [Escherichia coli O83:H1 str. LF82]	c_amazonense.gapfilled_200_mauve.fasta_1485
VFG035191(gi:387609951)	(EC042_4530) putative type VI secretion protein [SCI-I (SS183)] [Escherichia coli O44:H18 042]	c_amazonense.gapfilled_200_mauve.fasta_2297
VFG035228(gi:218696627)	(EC55989_3327) hypothetical protein [SCI-I T6SS (CVF734)] [Escherichia coli 55989]	c_amazonense.gapfilled_200_mauve.fasta_790
VFG035924(gi:387610457)	(aatC) ABC transporter ATP-binding protein AatC [ABC transporter for dispersin (CVF737)] [Escherichia coli O78:H11:K80 str. H10407]	c_amazonense.gapfilled_200_mauve.fasta_2108
VFG036164(gi:313668088)	(rfaF) ADP-heptose--LPS heptosyltransferase [LOS synthesis (CVF187)] [Neisseria lactamica 020-06]	c_amazonense.gapfilled_200_mauve.fasta_2231
VFG036325(gi:385341774)	(pilW) type IV pilus biogenesis/stability protein [Type IV pili (CVF189)] [Neisseria meningitidis M01-240149]	c_amazonense.gapfilled_200_mauve.fasta_1170
VFG036506(gi:385340887)	(porB) major outer membrane protein P.IB precursor [PorB (CVF195)] [Neisseria meningitidis G2136]	c_amazonense.gapfilled_200_mauve.fasta_1443
VFG036506(gi:385340887)	(porB) major outer membrane protein P.IB precursor [PorB (CVF195)] [Neisseria meningitidis G2136]	c_amazonense.gapfilled_200_mauve.fasta_2983
VFG036506(gi:385340887)	(porB) major outer membrane protein P.IB precursor [PorB (CVF195)] [Neisseria meningitidis G2136]	c_amazonense.gapfilled_200_mauve.fasta_3406
VFG036660(gi:313667887)	(exbD) biopolymer transport protein [Ton system (CVF202)] [Neisseria lactamica 020-06]	c_amazonense.gapfilled_200_mauve.fasta_289
VFG036660(gi:313667887)	(exbD) biopolymer transport protein [Ton system (CVF202)] [Neisseria lactamica 020-06]	c_amazonense.gapfilled_200_mauve.fasta_2919
VFG036785(gi:254804030)	(kdtA/waaA) 3-deoxy-D-manno-octulosonic-acid transferase [LOS synthesis (CVF187)] [Neisseria meningitidis alpha14]	c_amazonense.gapfilled_200_mauve.fasta_135
VFG036795(gi:218767265)	(rfaC) lipopolysaccharide heptosyltransferase I [LOS synthesis (CVF187)] [Neisseria meningitidis Z2491]	c_amazonense.gapfilled_200_mauve.fasta_136
VFG036909(gi:254804431)	(pilV) putative type IV pilin protein [Type IV pili (CVF189)] [Neisseria meningitidis alpha14]	c_amazonense.gapfilled_200_mauve.fasta_635
VFG036942(gi:194099567)	(farA) Efflux pump protein, fatty acid resistance [FarAB (CVF758)] [Neisseria gonorrhoeae NCCP11945]	c_amazonense.gapfilled_200_mauve.fasta_574
VFG036945(gi:254805646)	(farA) putative efflux pump protein [FarAB (CVF758)] [Neisseria meningitidis alpha14]	c_amazonense.gapfilled_200_mauve.fasta_619
VFG036954(gi:313667721)	(farA) multidrug resistance translocase [FarAB (CVF758)] [Neisseria lactamica 020-06]	c_amazonense.gapfilled_200_mauve.fasta_416
VFG036962(gi:385323459)	(farB) multidrug resistance protein B [FarAB (CVF758)] [Neisseria meningitidis 8013]	c_amazonense.gapfilled_200_mauve.fasta_3116
VFG036965(gi:385340745)	(farB) antibacterial fatty acid resistance protein B [FarAB (CVF758)] [Neisseria meningitidis G2136]	c_amazonense.gapfilled_200_mauve.fasta_573
VFG036972(gi:313667722)	(farB) multidrug resistance translocase [FarAB (CVF758)] [Neisseria lactamica 020-06]	c_amazonense.gapfilled_200_mauve.fasta_618
VFG036978(gi:194099138)	(mtrC) antibiotic resistance efflux pump component [MtrCDE (CVF759)] [Neisseria gonorrhoeae NCCP11945]	c_amazonense.gapfilled_200_mauve.fasta_1422
VFG036978(gi:194099138)	(mtrC) antibiotic resistance efflux pump component [MtrCDE (CVF759)] [Neisseria gonorrhoeae NCCP11945]	c_amazonense.gapfilled_200_mauve.fasta_324
VFG036987(gi:385855732)	(mtrC) membrane fusion protein MtrC [MtrCDE (CVF759)] [Neisseria meningitidis M01-240355]	c_amazonense.gapfilled_200_mauve.fasta_1498
VFG036991(gi:218768729)	(mtrD) drug efflux protein [MtrCDE (CVF759)] [Neisseria meningitidis Z2491]	c_amazonense.gapfilled_200_mauve.fasta_3412
VFG036997(gi:385338548)	(mtrD) drug efflux transport protein MtrD [MtrCDE (CVF759)] [Neisseria meningitidis WUE 2594]	c_amazonense.gapfilled_200_mauve.fasta_1421
VFG036998(gi:385323678)	(mtrD) drug efflux transport protein MtrD [MtrCDE (CVF759)] [Neisseria meningitidis 8013]	c_amazonense.gapfilled_200_mauve.fasta_3491
VFG037021(gi:385850751)	(mtrE) multiple transferable resistance system protein MtrE [MtrCDE (CVF759)] [Neisseria meningitidis M04-240196]	c_amazonense.gapfilled_200_mauve.fasta_322
VFG037047(gi:59800626)	(mntA) ABC transporter ATP-binding protein [Manganese transport system (CVF761)] [Neisseria gonorrhoeae FA 1090]	c_amazonense.gapfilled_200_mauve.fasta_2499
VFG037072(gi:385327921)	(mntB) putative ABC-transporter membrane protein [Manganese transport system (CVF761)] [Neisseria meningitidis alpha710]	c_amazonense.gapfilled_200_mauve.fasta_2498
VFG037089(gi:254804469)	(mntC) putative periplasmic solute binding protein [Manganese transport system (CVF761)] [Neisseria meningitidis alpha14]	c_amazonense.gapfilled_200_mauve.fasta_2497
VFG037105(gi:385337152)	(msrA/B(pilB)) peptide methionine sulfoxide reductase MsrA/MsrB [includes: thioredoxin, peptide methionine sulfoxide reductase MsrA (protein-methionine-S-oxide reductase	peptide N c_amazonense.gapfilled_200_mauve.fasta_2629
VFG037116(gi:313667375)	(msrA/B(pilB)) peptide methionine sulfoxide reductase [Methionine sulphoxide reductase (CVF762)] [Neisseria lactamica 020-06]	c_amazonense.gapfilled_200_mauve.fasta_2035
VFG037134(gi:313668782)	(recN) DNA repair protein [Recombinational repair protein (CVF763)] [Neisseria lactamica 020-06]	c_amazonense.gapfilled_200_mauve.fasta_2039
VFG037467(gi:384130865)	(ABK1_0905) transcriptional regulator [Heme utilization (CVF769)] [Acinetobacter baumannii 1656-2]	c_amazonense.gapfilled_200_mauve.fasta_3651
VFG037535(gi:407931896)	(hemO) heme oxygenase [Heme utilization (CVF769)] [Acinetobacter baumannii TYTH-1]	c_amazonense.gapfilled_200_mauve.fasta_3751
VFG037711(gi:469822479)	(adeF) RND efflux transporter [AdeFGH efflux pump/transport autoinducer (CVF773)] [Acinetobacter baumannii D1279779]	c_amazonense.gapfilled_200_mauve.fasta_3490
VFG037718(gi:213158705)	(adeG) RND family efflux transporter [AdeFGH efflux pump/transport autoinducer (CVF773)] [Acinetobacter baumannii AB0057]	c_amazonense.gapfilled_200_mauve.fasta_1499
VFG037718(gi:213158705)	(adeG) RND family efflux transporter [AdeFGH efflux pump/transport autoinducer (CVF773)] [Acinetobacter baumannii AB0057]	c_amazonense.gapfilled_200_mauve.fasta_2137
VFG037718(gi:213158705)	(adeG) RND family efflux transporter [AdeFGH efflux pump/transport autoinducer (CVF773)] [Acinetobacter baumannii AB0057]	c_amazonense.gapfilled_200_mauve.fasta_2138
VFG037718(gi:213158705)	(adeG) RND family efflux transporter [AdeFGH efflux pump/transport autoinducer (CVF773)] [Acinetobacter baumannii AB0057]	c_amazonense.gapfilled_200_mauve.fasta_323
VFG037741(gi:469822481)	(adeH) efflux pump outer membrane protein [AdeFGH efflux pump/transport autoinducer (CVF773)] [Acinetobacter baumannii D1279779]	c_amazonense.gapfilled_200_mauve.fasta_2139
VFG037745(gi:407933448)	(adeH) NodT family efflux transporter outer membrane lipoprotein [AdeFGH efflux pump/transport autoinducer (CVF773)] [Acinetobacter baumannii TYTH-1]	c_amazonense.gapfilled_200_mauve.fasta_413
VFG037745(gi:407933448)	(adeH) NodT family efflux transporter outer membrane lipoprotein [AdeFGH efflux pump/transport autoinducer (CVF773)] [Acinetobacter baumannii TYTH-1]	c_amazonense.gapfilled_200_mauve.fasta_575
VFG037745(gi:407933448)	(adeH) NodT family efflux transporter outer membrane lipoprotein [AdeFGH efflux pump/transport autoinducer (CVF773)] [Acinetobacter baumannii TYTH-1]	c_amazonense.gapfilled_200_mauve.fasta_620
VFG037766(gi:523529591)	(lpxL) Lauroyl/myristoyl acyltransferase [LPS (CVF774)] [Acinetobacter baumannii BJAB0715]	c_amazonense.gapfilled_200_mauve.fasta_846
VFG037923(gi:407930969)	(M3Q_285) nucleoside-diphosphate sugar epimerase [Capsule (CVF775)] [Acinetobacter baumannii TYTH-1]	c_amazonense.gapfilled_200_mauve.fasta_3519
VFG037928(gi:126640167)	(A1S_0054) WbbJ protein [Capsule (CVF775)] [Acinetobacter baumannii ATCC 17978]	c_amazonense.gapfilled_200_mauve.fasta_3619
VFG037958(gi:126640169)	(A1S_0056) O-antigen translocase [Capsule (CVF775)] [Acinetobacter baumannii ATCC 17978]	c_amazonense.gapfilled_200_mauve.fasta_3617
VFG037983(gi:407930973)	(M3Q_289) Sel1 repeat protein [Capsule (CVF775)] [Acinetobacter baumannii TYTH-1]	c_amazonense.gapfilled_200_mauve.fasta_3396
VFG037990(gi:523521483)	(BJAB07104_00096) putative UDP-glucose 6-dehydrogenase [Capsule (CVF775)] [Acinetobacter baumannii BJAB07104]	c_amazonense.gapfilled_200_mauve.fasta_2477
VFG037996(gi:169632089)	(ABSDF0072) polysaccharide biosynthesis protein [Capsule (CVF775)] [Acinetobacter baumannii SDF]	c_amazonense.gapfilled_200_mauve.fasta_3727
VFG038074(gi:384130057)	(ABK1_0097) transposase [Capsule (CVF775)] [Acinetobacter baumannii 1656-2]	c_amazonense.gapfilled_200_mauve.fasta_1121
VFG038074(gi:384130057)	(ABK1_0097) transposase [Capsule (CVF775)] [Acinetobacter baumannii 1656-2]	c_amazonense.gapfilled_200_mauve.fasta_1196
VFG038074(gi:384130057)	(ABK1_0097) transposase [Capsule (CVF775)] [Acinetobacter baumannii 1656-2]	c_amazonense.gapfilled_200_mauve.fasta_1305
VFG038074(gi:384130057)	(ABK1_0097) transposase [Capsule (CVF775)] [Acinetobacter baumannii 1656-2]	c_amazonense.gapfilled_200_mauve.fasta_1486
VFG038074(gi:384130057)	(ABK1_0097) transposase [Capsule (CVF775)] [Acinetobacter baumannii 1656-2]	c_amazonense.gapfilled_200_mauve.fasta_1487
VFG038074(gi:384130057)	(ABK1_0097) transposase [Capsule (CVF775)] [Acinetobacter baumannii 1656-2]	c_amazonense.gapfilled_200_mauve.fasta_1716
VFG038074(gi:384130057)	(ABK1_0097) transposase [Capsule (CVF775)] [Acinetobacter baumannii 1656-2]	c_amazonense.gapfilled_200_mauve.fasta_1866
VFG038074(gi:384130057)	(ABK1_0097) transposase [Capsule (CVF775)] [Acinetobacter baumannii 1656-2]	c_amazonense.gapfilled_200_mauve.fasta_3942
VFG038074(gi:384130057)	(ABK1_0097) transposase [Capsule (CVF775)] [Acinetobacter baumannii 1656-2]	c_amazonense.gapfilled_200_mauve.fasta_4031
VFG038074(gi:384130057)	(ABK1_0097) transposase [Capsule (CVF775)] [Acinetobacter baumannii 1656-2]	c_amazonense.gapfilled_200_mauve.fasta_4146
VFG038074(gi:384130057)	(ABK1_0097) transposase [Capsule (CVF775)] [Acinetobacter baumannii 1656-2]	c_amazonense.gapfilled_200_mauve.fasta_4151
VFG038074(gi:384130057)	(ABK1_0097) transposase [Capsule (CVF775)] [Acinetobacter baumannii 1656-2]	c_amazonense.gapfilled_200_mauve.fasta_687
VFG038074(gi:384130057)	(ABK1_0097) transposase [Capsule (CVF775)] [Acinetobacter baumannii 1656-2]	c_amazonense.gapfilled_200_mauve.fasta_689
VFG038074(gi:384130057)	(ABK1_0097) transposase [Capsule (CVF775)] [Acinetobacter baumannii 1656-2]	c_amazonense.gapfilled_200_mauve.fasta_765
VFG038216(gi:384130704)	(bfmR) putative response regulator (activator) in two-component regulatory system (OmpR family) [Two-component system (CVF778)] [Acinetobacter baumannii 1656-2]	c_amazonense.gapfilled_200_mauve.fasta_2537
VFG038216(gi:384130704)	(bfmR) putative response regulator (activator) in two-component regulatory system (OmpR family) [Two-component system (CVF778)] [Acinetobacter baumannii 1656-2]	c_amazonense.gapfilled_200_mauve.fasta_3418
VFG038216(gi:384130704)	(bfmR) putative response regulator (activator) in two-component regulatory system (OmpR family) [Two-component system (CVF778)] [Acinetobacter baumannii 1656-2]	c_amazonense.gapfilled_200_mauve.fasta_612
VFG038240(gi:469820883)	(bfmS) sensory histidine kinase in two-component regulatory system with RstA [Two-component system (CVF778)] [Acinetobacter baumannii D1279779]	c_amazonense.gapfilled_200_mauve.fasta_3419
VFG038245(gi:407931632)	(bfmS) signal transduction histidine kinase [Two-component system (CVF778)] [Acinetobacter baumannii TYTH-1]	c_amazonense.gapfilled_200_mauve.fasta_611
VFG038250(gi:126640330)	(pbpG) D-alanyl-D-alanine endopeptidase [PbpG (CVF779)] [Acinetobacter baumannii ATCC 17978]	c_amazonense.gapfilled_200_mauve.fasta_2577
VFG038417(gi:507520054)	(fimD) fimbrial chaperone protein [Type I pili (VF0486)] [Aeromonas hydrophila ML09-119]	c_amazonense.gapfilled_200_mauve.fasta_3123
VFG038481(gi:145297759)	(tppB) type IV pilus assembly protein [Tap type IV pili (CVF783)] [Aeromonas salmonicida subsp. salmonicida A449]	c_amazonense.gapfilled_200_mauve.fasta_2542
VFG038722(gi:117620880)	(AHA_1389) CobQ/CobB/MinD/ParA family protein [Polar flagella (CVF786)] [Aeromonas hydrophila subsp. hydrophila ATCC 7966]	c_amazonense.gapfilled_200_mauve.fasta_364
VFG038724(gi:145298370)	(ASA_1361) SOJ-like and chromosome partitioning protein [Polar flagella (CVF786)] [Aeromonas salmonicida subsp. salmonicida A449]	c_amazonense.gapfilled_200_mauve.fasta_4178
VFG038724(gi:145298370)	(ASA_1361) SOJ-like and chromosome partitioning protein [Polar flagella (CVF786)] [Aeromonas salmonicida subsp. salmonicida A449]	c_amazonense.gapfilled_200_mauve.fasta_540
VFG038731(gi:507521385)	(flaA) flagellin [Polar flagella (VF0473)] [Aeromonas hydrophila ML09-119]	c_amazonense.gapfilled_200_mauve.fasta_1798
VFG038731(gi:507521385)	(flaA) flagellin [Polar flagella (VF0473)] [Aeromonas hydrophila ML09-119]	c_amazonense.gapfilled_200_mauve.fasta_3436
VFG038817(gi:145298486)	(cheV) two-component system chemotaxis response regulator CheV [Polar flagella (CVF786)] [Aeromonas salmonicida subsp. salmonicida A449]	c_amazonense.gapfilled_200_mauve.fasta_2998
VFG038839(gi:117621307)	(flmH) short chain dehydrogenase/reductase family oxidoreductase [Polar flagella (CVF786)] [Aeromonas hydrophila subsp. hydrophila ATCC 7966]	c_amazonense.gapfilled_200_mauve.fasta_1378
VFG038839(gi:117621307)	(flmH) short chain dehydrogenase/reductase family oxidoreductase [Polar flagella (CVF786)] [Aeromonas hydrophila subsp. hydrophila ATCC 7966]	c_amazonense.gapfilled_200_mauve.fasta_2279
VFG038840(gi:507521851)	(flmH) 3-oxoacyl-ACP reductase [Polar flagella (VF0473)] [Aeromonas hydrophila ML09-119]	c_amazonense.gapfilled_200_mauve.fasta_1991
VFG038840(gi:507521851)	(flmH) 3-oxoacyl-ACP reductase [Polar flagella (VF0473)] [Aeromonas hydrophila ML09-119]	c_amazonense.gapfilled_200_mauve.fasta_2082
VFG038840(gi:507521851)	(flmH) 3-oxoacyl-ACP reductase [Polar flagella (VF0473)] [Aeromonas hydrophila ML09-119]	c_amazonense.gapfilled_200_mauve.fasta_2310
VFG038840(gi:507521851)	(flmH) 3-oxoacyl-ACP reductase [Polar flagella (VF0473)] [Aeromonas hydrophila ML09-119]	c_amazonense.gapfilled_200_mauve.fasta_2973
VFG038840(gi:507521851)	(flmH) 3-oxoacyl-ACP reductase [Polar flagella (VF0473)] [Aeromonas hydrophila ML09-119]	c_amazonense.gapfilled_200_mauve.fasta_3101
VFG038840(gi:507521851)	(flmH) 3-oxoacyl-ACP reductase [Polar flagella (VF0473)] [Aeromonas hydrophila ML09-119]	c_amazonense.gapfilled_200_mauve.fasta_3625
VFG038840(gi:507521851)	(flmH) 3-oxoacyl-ACP reductase [Polar flagella (VF0473)] [Aeromonas hydrophila ML09-119]	c_amazonense.gapfilled_200_mauve.fasta_568
VFG038841(gi:145297258)	(flmH) 3-oxoacyl-ACP reductase [Polar flagella (CVF786)] [Aeromonas salmonicida subsp. salmonicida A449]	c_amazonense.gapfilled_200_mauve.fasta_3321
VFG038859(gi:145297439)	(lfiR) lateral flagellar biosynthetic protein LfiR [Lateral flagella (VF0474)] [Aeromonas salmonicida subsp. salmonicida A449]	c_amazonense.gapfilled_200_mauve.fasta_2446
VFG038902(gi:145298519)	(hlyA) hemolysin [Hemolysin, HlyA (CVF792)] [Aeromonas salmonicida subsp. salmonicida A449]	c_amazonense.gapfilled_200_mauve.fasta_140
VFG038903(gi:330830274)	(hlyA) putative transporter [Hemolysin, HlyA (CVF792)] [Aeromonas veronii B565]	c_amazonense.gapfilled_200_mauve.fasta_3741
VFG038915(gi:117619233)	(rtxH) hypothetical protein [The repeat in toxin (RTX) (CVF795)] [Aeromonas hydrophila subsp. hydrophila ATCC 7966]	c_amazonense.gapfilled_200_mauve.fasta_1293
VFG038917(gi:117618037)	(rtxD) RTX toxin transporter [The repeat in toxin (RTX) (CVF795)] [Aeromonas hydrophila subsp. hydrophila ATCC 7966]	c_amazonense.gapfilled_200_mauve.fasta_1303
VFG038936(gi:88607176)	(virB4-1) type IV secretion system ATPase VirB4 [Rvh T4SS (VF0487)] [Anaplasma phagocytophilum HZ]	c_amazonense.gapfilled_200_mauve.fasta_4262
VFG039032(gi:88607229)	(virB11) type IV secretion system ATPase VirB11 [Rvh T4SS (VF0487)] [Anaplasma phagocytophilum HZ]	c_amazonense.gapfilled_200_mauve.fasta_4270
VFG039383(gi:212218285)	(coxH2/rimL) ribosomal-protein-S18-alanine acetyltransferase [T4SS effectors (CVF803)] [Coxiella burnetii CbuK_Q154]	c_amazonense.gapfilled_200_mauve.fasta_3004
VFG039427(gi:212218523)	(CbuK_0945) hypothetical protein [T4SS effectors (CVF803)] [Coxiella burnetii CbuK_Q154]	c_amazonense.gapfilled_200_mauve.fasta_2475
VFG039489(gi:154706898)	(CBUD_0561) hypothetical protein [T4SS effectors (CVF803)] [Coxiella burnetii Dugway 5J108-111]	c_amazonense.gapfilled_200_mauve.fasta_1286
VFG039532(gi:212212082)	(CbuG_0446) hypothetical protein [T4SS effectors (CVF803)] [Coxiella burnetii CbuG_Q212]	c_amazonense.gapfilled_200_mauve.fasta_2553
VFG039546(gi:212219305)	(CbuK_1823) GatB/Yqey domain protein [T4SS effectors (CVF803)] [Coxiella burnetii CbuK_Q154]	c_amazonense.gapfilled_200_mauve.fasta_3341
VFG039611(gi:212211810)	(coxH3) alpha/beta hydrolase [T4SS effectors (CVF803)] [Coxiella burnetii CbuG_Q212]	c_amazonense.gapfilled_200_mauve.fasta_1803
VFG040782(gi:238650849)	(pld) phospholipase D superfamily protein PLD [Phospholipase D (CVF814)] [Rickettsia peacockii str. Rustic]	c_amazonense.gapfilled_200_mauve.fasta_4292
VFG040920(gi:53717647)	(gspE) general secretory pathway protein E [gsp (SS210)] [Burkholderia pseudomallei K96243]	c_amazonense.gapfilled_200_mauve.fasta_3390
VFG040921(gi:53717648)	(gspF) general secretory pathway protein F [gsp (SS210)] [Burkholderia pseudomallei K96243]	c_amazonense.gapfilled_200_mauve.fasta_3389
VFG040927(gi:53717654)	(gspK) general secretory pathway protein K [gsp (SS210)] [Burkholderia pseudomallei K96243]	c_amazonense.gapfilled_200_mauve.fasta_3384
VFG040930(gi:53717657)	(gspN) general secretory pathway protein N [gsp (SS210)] [Burkholderia pseudomallei K96243]	c_amazonense.gapfilled_200_mauve.fasta_3381
VFG040973(gi:17547833)	(gspD) general secretory pathway D transmembrane protein [gsp (SS217)] [Ralstonia solanacearum GMI1000]	c_amazonense.gapfilled_200_mauve.fasta_3391
VFG040978(gi:17547827)	(gspI) general secretory pathway I transmembrane protein [gsp (SS217)] [Ralstonia solanacearum GMI1000]	c_amazonense.gapfilled_200_mauve.fasta_3386
VFG040979(gi:17547825)	(gspG) putative general secretion pathway G transmembrane protein [gsp (SS217)] [Ralstonia solanacearum GMI1000]	c_amazonense.gapfilled_200_mauve.fasta_3388
VFG041012(gi:15596708)	(vgrG2) hypothetical protein [HSI-2 (SS179)] [Pseudomonas aeruginosa PAO1]	c_amazonense.gapfilled_200_mauve.fasta_1494
VFG041012(gi:15596708)	(vgrG2) hypothetical protein [HSI-2 (SS179)] [Pseudomonas aeruginosa PAO1]	c_amazonense.gapfilled_200_mauve.fasta_1686
VFG041012(gi:15596708)	(vgrG2) hypothetical protein [HSI-2 (SS179)] [Pseudomonas aeruginosa PAO1]	c_amazonense.gapfilled_200_mauve.fasta_3641
VFG041012(gi:15596708)	(vgrG2) hypothetical protein [HSI-2 (SS179)] [Pseudomonas aeruginosa PAO1]	c_amazonense.gapfilled_200_mauve.fasta_783
VFG041139(gi:206558740)	(bscS) periplasmic solute-binding protein [T6SS (SS193)] [Burkholderia cenocepacia J2315]	c_amazonense.gapfilled_200_mauve.fasta_2423
VFG041139(gi:206558740)	(bscS) periplasmic solute-binding protein [T6SS (SS193)] [Burkholderia cenocepacia J2315]	c_amazonense.gapfilled_200_mauve.fasta_3551

VFG041139(gi:206558740)	(bscS) periplasmic solute-binding protein [T6SS (SS193)] [Burkholderia cenocepacia J2315]	c_amazonense.gapfilled_200_mauve.fasta_99
VFG041142(gi:206558743)	(tssL/bscP) putative type VI secretion system protein TssL [T6SS (SS193)] [Burkholderia cenocepacia J2315]	c_amazonense.gapfilled_200_mauve.fasta_773
VFG041143(gi:206558744)	(tssK/bscO) putative type VI secretion system protein TssK [T6SS (SS193)] [Burkholderia cenocepacia J2315]	c_amazonense.gapfilled_200_mauve.fasta_774
VFG041146(gi:206558747)	(tssB/bscL) putative type VI secretion system protein TssB [T6SS (SS193)] [Burkholderia cenocepacia J2315]	c_amazonense.gapfilled_200_mauve.fasta_779
VFG041147(gi:206558748)	(tssC/bscK) putative type VI secretion system protein TssC [T6SS (SS193)] [Burkholderia cenocepacia J2315]	c_amazonense.gapfilled_200_mauve.fasta_780
VFG041149(gi:206558750)	(tssE/bscI) putative type VI secretion system protein TssE [T6SS (SS193)] [Burkholderia cenocepacia J2315]	c_amazonense.gapfilled_200_mauve.fasta_772
VFG041156(gi:206558757)	(tssM/icmF/bscB) putative type VI secretion system protein TssM [T6SS (SS193)] [Burkholderia cenocepacia J2315]	c_amazonense.gapfilled_200_mauve.fasta_777
VFG041184(gi:269139780)	(evpG) type VI secretion system protein EvpG [EVP (E. tarda virulent protein) (SS189)] [Edwardsiella tarda EIB202]	c_amazonense.gapfilled_200_mauve.fasta_786
VFG041188(gi:269139784)	(evpK) type VI secretion system protein EvpK [EVP (E. tarda virulent protein) (SS189)] [Edwardsiella tarda EIB202]	c_amazonense.gapfilled_200_mauve.fasta_791
VFG041304(gb|YP_095978)	(lirB) Dot/Icm type IV secretion system effector LirB [Dot/Icm (SS047)] [Legionella pneumophila subsp. pneumophila str. Philadelphia 1]	c_amazonense.gapfilled_200_mauve.fasta_2600
VFG041304(gb|YP_095978)	(lirB) Dot/Icm type IV secretion system effector LirB [Dot/Icm (SS047)] [Legionella pneumophila subsp. pneumophila str. Philadelphia 1]	c_amazonense.gapfilled_200_mauve.fasta_2601
VFG041371(gi:88608055)	(virB9-1) type IV secretion system protein VirB9 [T4SS (SS055)] [Neorickettsia sennetsu str. Miyayama]	c_amazonense.gapfilled_200_mauve.fasta_4268
VFG041571(gi:283784668)	(nleK) hypothetical protein [T3SS (SS022)] [Citrobacter rodentium ICC168]	c_amazonense.gapfilled_200_mauve.fasta_302
VFG041575(gi:269138242)	(esrB) two-component response regulator EsrB, LuxR family [T3SS (SS025)] [Edwardsiella tarda EIB202]	c_amazonense.gapfilled_200_mauve.fasta_2666
VFG041577(gi:269138241)	(esrA) two-component sensor/regulator EsrA [T3SS (SS025)] [Edwardsiella tarda EIB202]	c_amazonense.gapfilled_200_mauve.fasta_2667
VFG041578(gi:269138240)	(ETAE_0884) putative transglycosylase signal peptide protein [T3SS (SS025)] [Edwardsiella tarda EIB202]	c_amazonense.gapfilled_200_mauve.fasta_2668
VFG041579(gi:269138239)	(esaU) type III secretion apparatus protein [T3SS (SS025)] [Edwardsiella tarda EIB202]	c_amazonense.gapfilled_200_mauve.fasta_2669
VFG041580(gi:269138237)	(esaS) type III secretion apparatus [T3SS (SS025)] [Edwardsiella tarda EIB202]	c_amazonense.gapfilled_200_mauve.fasta_2671
VFG041581(gi:269138238)	(esaT) type III secretion apparatus protein [T3SS (SS025)] [Edwardsiella tarda EIB202]	c_amazonense.gapfilled_200_mauve.fasta_2670
VFG041582(gi:269138235)	(esaM) type III secretion apparatus protein [T3SS (SS025)] [Edwardsiella tarda EIB202]	c_amazonense.gapfilled_200_mauve.fasta_2675
VFG041583(gi:269138236)	(esaR) type three secretion apparatus protein R [T3SS (SS025)] [Edwardsiella tarda EIB202]	c_amazonense.gapfilled_200_mauve.fasta_2672
VFG041584(gi:269138233)	(esaN) type III secretion system ATPase [T3SS (SS025)] [Edwardsiella tarda EIB202]	c_amazonense.gapfilled_200_mauve.fasta_2677
VFG041585(gi:269138234)	(esaV) type III secretion protein, HrcV family [T3SS (SS025)] [Edwardsiella tarda EIB202]	c_amazonense.gapfilled_200_mauve.fasta_2676
VFG041586(gi:269138230)	(esaQ) translocation protein in type III secretion [T3SS (SS025)] [Edwardsiella tarda EIB202]	c_amazonense.gapfilled_200_mauve.fasta_2680
VFG041587(gi:269138231)	(esaP) putative major facilitator family transporter [T3SS (SS025)] [Edwardsiella tarda EIB202]	c_amazonense.gapfilled_200_mauve.fasta_2679
VFG041588(gi:269138232)	(esaO) type III secretion system ATPase [T3SS (SS025)] [Edwardsiella tarda EIB202]	c_amazonense.gapfilled_200_mauve.fasta_2678
VFG041589(gi:269138227)	(escA) type III secretion low calcium response chaperone [T3SS (SS025)] [Edwardsiella tarda EIB202]	c_amazonense.gapfilled_200_mauve.fasta_2684
VFG041590(gi:269138228)	(eseB) EspA family secreted protein [T3SS (SS025)] [Edwardsiella tarda EIB202]	c_amazonense.gapfilled_200_mauve.fasta_2682
VFG041590(gi:269138228)	(eseB) EspA family secreted protein [T3SS (SS025)] [Edwardsiella tarda EIB202]	c_amazonense.gapfilled_200_mauve.fasta_2683
VFG041591(gi:269138229)	(escC) hypothetical protein [T3SS (SS025)] [Edwardsiella tarda EIB202]	c_amazonense.gapfilled_200_mauve.fasta_2681
VFG041592(gi:269138224)	(eseE) type III secretion system effector protein E [T3SS (SS025)] [Edwardsiella tarda EIB202]	c_amazonense.gapfilled_200_mauve.fasta_2687
VFG041593(gi:269138225)	(eseD) type III secretion system effector protein D [T3SS (SS025)] [Edwardsiella tarda EIB202]	c_amazonense.gapfilled_200_mauve.fasta_2686
VFG041594(gi:269138226)	(eseC) type III secretion system effector protein C [T3SS (SS025)] [Edwardsiella tarda EIB202]	c_amazonense.gapfilled_200_mauve.fasta_2685
VFG041595(gi:269138223)	(escB) type III secretion system chaperone protein B [T3SS (SS025)] [Edwardsiella tarda EIB202]	c_amazonense.gapfilled_200_mauve.fasta_2689
VFG041596(gi:269138221)	(esaB) hypothetical protein [T3SS (SS025)] [Edwardsiella tarda EIB202]	c_amazonense.gapfilled_200_mauve.fasta_2665
VFG041598(gi:269138220)	(esaC) Type II secretory pathway, component PulD like protein [T3SS (SS025)] [Edwardsiella tarda EIB202]	c_amazonense.gapfilled_200_mauve.fasta_2664
VFG041599(gi:269138218)	(ETAE_0862) hypothetical protein [T3SS (SS025)] [Edwardsiella tarda EIB202]	c_amazonense.gapfilled_200_mauve.fasta_2662
VFG041600(gi:269138219)	(esaD) type-III secretion protein [T3SS (SS025)] [Edwardsiella tarda EIB202]	c_amazonense.gapfilled_200_mauve.fasta_2663
VFG041601(gi:269138213)	(esaJ) putative type III secretion system apparatus lipoprotein [T3SS (SS025)] [Edwardsiella tarda EIB202]	c_amazonense.gapfilled_200_mauve.fasta_2657
VFG041602(gi:269138214)	(esaI) putative type III secretion apparatus [T3SS (SS025)] [Edwardsiella tarda EIB202]	c_amazonense.gapfilled_200_mauve.fasta_2658
VFG041604(gi:269138216)	(esaG) putative type III secretion system needle protein [T3SS (SS025)] [Edwardsiella tarda EIB202]	c_amazonense.gapfilled_200_mauve.fasta_2660
VFG041605(gi:269138217)	(esrC) putative transcriptional regulator EsrC [T3SS (SS025)] [Edwardsiella tarda EIB202]	c_amazonense.gapfilled_200_mauve.fasta_2661
VFG041606(gi:269138212)	(ETAE_0856) hypothetical protein [T3SS (SS025)] [Edwardsiella tarda EIB202]	c_amazonense.gapfilled_200_mauve.fasta_2656
VFG041607(gi:269138211)	(esaK) putative type III secretion apparatus [T3SS (SS025)] [Edwardsiella tarda EIB202]	c_amazonense.gapfilled_200_mauve.fasta_2655
VFG041609(gi:269138210)	(esaL) putative type III secretion apparatus [T3SS (SS025)] [Edwardsiella tarda EIB202]	c_amazonense.gapfilled_200_mauve.fasta_2654
VFG041721(gi:206563884)	(virB1) type III secretion system protein [T3SS (SS015)] [Burkholderia cenocepacia J2315]	c_amazonense.gapfilled_200_mauve.fasta_4209
VFG041722(gi:206563885)	(BCAM2044) putative asparagine synthetase [T3SS (SS015)] [Burkholderia cenocepacia J2315]	c_amazonense.gapfilled_200_mauve.fasta_650
VFG041839(gi:34498095)	(cilA) invasion protein regulator [Cpi-1a + Cpi-1 (SPI-1 like) (SS016)] [Chromobacterium violaceum ATCC 12472]	c_amazonense.gapfilled_200_mauve.fasta_1878
VFG041842(gi:34498090)	(armR) two-component response regulator [Cpi-1a + Cpi-1 (SPI-1 like) (SS016)] [Chromobacterium violaceum ATCC 12472]	c_amazonense.gapfilled_200_mauve.fasta_734
VFG041843(gi:34498093)	(CV_2638) hypothetical protein [Cpi-1a + Cpi-1 (SPI-1 like) (SS016)] [Chromobacterium violaceum ATCC 12472]	c_amazonense.gapfilled_200_mauve.fasta_1873
VFG041844(gi:34498087)	(civG) invasion protein - outer membrane [Cpi-1a + Cpi-1 (SPI-1 like) (SS016)] [Chromobacterium violaceum ATCC 12472]	c_amazonense.gapfilled_200_mauve.fasta_1870
VFG041845(gi:34498086)	(civE) invasion protein [Cpi-1a + Cpi-1 (SPI-1 like) (SS016)] [Chromobacterium violaceum ATCC 12472]	c_amazonense.gapfilled_200_mauve.fasta_1869
VFG041846(gi:34498085)	(civA) invasion protein [Cpi-1a + Cpi-1 (SPI-1 like) (SS016)] [Chromobacterium violaceum ATCC 12472]	c_amazonense.gapfilled_200_mauve.fasta_1853
VFG041847(gi:34498084)	(civB) surface presentation of antigens, secretory protein [Cpi-1a + Cpi-1 (SPI-1 like) (SS016)] [Chromobacterium violaceum ATCC 12472]	c_amazonense.gapfilled_200_mauve.fasta_1852
VFG041849(gi:34498083)	(civC) ATP synthase SpaL [Cpi-1a + Cpi-1 (SPI-1 like) (SS016)] [Chromobacterium violaceum ATCC 12472]	c_amazonense.gapfilled_200_mauve.fasta_1851
VFG041852(gi:34498079)	(cpaP) surface presentation of antigens protein SpaP [Cpi-1a + Cpi-1 (SPI-1 like) (SS016)] [Chromobacterium violaceum ATCC 12472]	c_amazonense.gapfilled_200_mauve.fasta_1847
VFG041853(gi:34498076)	(cpaS) surface presentation of antigens protein SpaS [Cpi-1a + Cpi-1 (SPI-1 like) (SS016)] [Chromobacterium violaceum ATCC 12472]	c_amazonense.gapfilled_200_mauve.fasta_1844
VFG041854(gi:34498077)	(cpaR) surface presentation of antigens secretory protein [Cpi-1a + Cpi-1 (SPI-1 like) (SS016)] [Chromobacterium violaceum ATCC 12472]	c_amazonense.gapfilled_200_mauve.fasta_1845
VFG041855(gi:34498078)	(cpaQ) surface presentation of antigens secretory protein [Cpi-1a + Cpi-1 (SPI-1 like) (SS016)] [Chromobacterium violaceum ATCC 12472]	c_amazonense.gapfilled_200_mauve.fasta_1846
VFG041856(gi:34498075)	(cicA) surface presentation of antigens secretory protein [Cpi-1a + Cpi-1 (SPI-1 like) (SS016)] [Chromobacterium violaceum ATCC 12472]	c_amazonense.gapfilled_200_mauve.fasta_1843
VFG041857(gi:34498074)	(cipB) cell invasion protein [Cpi-1a + Cpi-1 (SPI-1 like) (SS016)] [Chromobacterium violaceum ATCC 12472]	c_amazonense.gapfilled_200_mauve.fasta_1842
VFG041881(gi:34498047)	(cpbD) carbohydrate-binding protein [Cpi-2 encoded T3SS (SPI-2 like) (SS023)] [Chromobacterium violaceum ATCC 12472]	c_amazonense.gapfilled_200_mauve.fasta_443
VFG041902(gi:34497876)	(cprJ) cell invasion protein - cytoplasmic [Cpi-1a + Cpi-1 (SPI-1 like) (SS016)] [Chromobacterium violaceum ATCC 12472]	c_amazonense.gapfilled_200_mauve.fasta_1720
VFG042099(gi:13475322)	(mlr6361) hypothetical protein [T3SS (SS026)] [Mesorhizobium loti MAFF303099]	c_amazonense.gapfilled_200_mauve.fasta_673
VFG042228(gi:82470754)	(ABB77406) unknown [T3SS (SS007)] [Pantoea stewartii subsp. stewartii str. SS104]	c_amazonense.gapfilled_200_mauve.fasta_1260
VFG042313(gi:16763947)	(STM0570) outer membrane esterase [ApeE (SS104)] [Salmonella enterica subsp. enterica serovar Typhimurium str. LT2]	c_amazonense.gapfilled_200_mauve.fasta_882
VFG042316(gi:34497372)	(CV_1917) hemolysin activator protein [ShlA-ShlB (SS143)] [Chromobacterium violaceum ATCC 12472]	c_amazonense.gapfilled_200_mauve.fasta_2325
VFG042317(gi:34497373)	(CV_1918) hemolysin [ShlA-ShlB (SS143)] [Chromobacterium violaceum ATCC 12472]	c_amazonense.gapfilled_200_mauve.fasta_2324
VFG042569(gi:26247121)	(focX) putative regulatory protein [F1C pili (AI043)] [Escherichia coli CFT073]	c_amazonense.gapfilled_200_mauve.fasta_621
VFG042619(gi:197284434)	(ucaA) major fimbrial subunit [uroepithelial cell adhesin (UCA) (AI071)] [Proteus mirabilis HI4320]	c_amazonense.gapfilled_200_mauve.fasta_3130
VFG042633(gi:15042709)	(mrfC) MrfC [mannose-resistant fimbriae, mrf (AI073)] [Photorhabdus luminescens str. K122]	c_amazonense.gapfilled_200_mauve.fasta_3122
VFG042701(gi:15599279)	(cupB3) usher CupB3 [CupB fimbriae (AI086)] [Pseudomonas aeruginosa PAO1]	c_amazonense.gapfilled_200_mauve.fasta_3128
VFG042736(gi:15599658)	(rpoN) RNA polymerase factor sigma-54 [type IV pili (AI097)] [Pseudomonas aeruginosa PAO1]	c_amazonense.gapfilled_200_mauve.fasta_2904
VFG042739(gi:15600525)	(crc) catabolite repression control protein [type IV pili (AI097)] [Pseudomonas aeruginosa PAO1]	c_amazonense.gapfilled_200_mauve.fasta_3746
VFG042789(gi:215276199)	(bfpD) nucleotide binding protein [bundle-forming pili, Bfp (AI101)] [Escherichia coli O127:H6 str. E2348/69]	c_amazonense.gapfilled_200_mauve.fasta_1861
VFG042931(gi:146329004)	(ppk) polyphosphate kinase [type IV pili (AI117)] [Dichelobacter nodosus VCS1703A]	c_amazonense.gapfilled_200_mauve.fasta_2928
VFG042955(gi:28199570)	(pilM) fimbrial assembly membrane protein [type IV pili (AI118)] [Xylella fastidiosa Temecula1]	c_amazonense.gapfilled_200_mauve.fasta_678
VFG043018(gi:15598545)	(cheV) chemotaxis protein [Deoxyhexose linking sugar, 209 Da capping structure (AI138)] [Pseudomonas aeruginosa PAO1]	c_amazonense.gapfilled_200_mauve.fasta_2472
VFG043018(gi:15598545)	(cheV) chemotaxis protein [Deoxyhexose linking sugar, 209 Da capping structure (AI138)] [Pseudomonas aeruginosa PAO1]	c_amazonense.gapfilled_200_mauve.fasta_2997
VFG043028(gi:16764536)	(flgH) flagellar basal body L-ring protein [peritrichous flagella (AI139)] [Salmonella enterica subsp. enterica serovar Typhimurium str. LT2]	c_amazonense.gapfilled_200_mauve.fasta_2355
VFG043039(gi:16765260)	(cheR) chemotaxis methyltransferase CheR [peritrichous flagella (AI139)] [Salmonella enterica subsp. enterica serovar Typhimurium str. LT2]	c_amazonense.gapfilled_200_mauve.fasta_1810
VFG043041(gi:16765262)	(cheW) purine-binding chemotaxis protein [peritrichous flagella (AI139)] [Salmonella enterica subsp. enterica serovar Typhimurium str. LT2]	c_amazonense.gapfilled_200_mauve.fasta_1811
VFG043047(gi:16765292)	(fliY) cystine transporter subunit [peritrichous flagella (AI139)] [Salmonella enterica subsp. enterica serovar Typhimurium str. LT2]	c_amazonense.gapfilled_200_mauve.fasta_2518
VFG043047(gi:16765292)	(fliY) cystine transporter subunit [peritrichous flagella (AI139)] [Salmonella enterica subsp. enterica serovar Typhimurium str. LT2]	c_amazonense.gapfilled_200_mauve.fasta_711
VFG043066(gi:16765317)	(fliP) flagellar biosynthesis protein FliP [peritrichous flagella (AI139)] [Salmonella enterica subsp. enterica serovar Typhimurium str. LT2]	c_amazonense.gapfilled_200_mauve.fasta_2557
VFG043099(gi:15802355)	(fliY) cystine transporter subunit [peritrichous flagella (AI140)] [Escherichia coli O157:H7 str. EDL933]	c_amazonense.gapfilled_200_mauve.fasta_2114
VFG043099(gi:15802355)	(fliY) cystine transporter subunit [peritrichous flagella (AI140)] [Escherichia coli O157:H7 str. EDL933]	c_amazonense.gapfilled_200_mauve.fasta_2931
VFG043099(gi:15802355)	(fliY) cystine transporter subunit [peritrichous flagella (AI140)] [Escherichia coli O157:H7 str. EDL933]	c_amazonense.gapfilled_200_mauve.fasta_3146
VFG043103(gi:15802359)	(fliD) flagellar capping protein [peritrichous flagella (AI140)] [Escherichia coli O157:H7 str. EDL933]	c_amazonense.gapfilled_200_mauve.fasta_1778
VFG043113(gi:15802379)	(fliL) flagellar basal body-associated protein FliL [peritrichous flagella (AI140)] [Escherichia coli O157:H7 str. EDL933]	c_amazonense.gapfilled_200_mauve.fasta_2561
VFG043131(gi:28900123)	(flgF) putative flagellar basal-body rod protein [lateral flagella (AI142)] [Vibrio parahaemolyticus RIMD 2210633]	c_amazonense.gapfilled_200_mauve.fasta_2809
VFG043133(gi:28900125)	(flgH) flagellar basal body L-ring protein [lateral flagella (AI142)] [Vibrio parahaemolyticus RIMD 2210633]	c_amazonense.gapfilled_200_mauve.fasta_2807
VFG043136(gi:28900128)	(flgK) putative flagellar hook-associated protein [lateral flagella (AI142)] [Vibrio parahaemolyticus RIMD 2210633]	c_amazonense.gapfilled_200_mauve.fasta_2804
VFG043137(gi:28900129)	(flgL) flagellar hook-associated protein FlgL [lateral flagella (AI142)] [Vibrio parahaemolyticus RIMD 2210633]	c_amazonense.gapfilled_200_mauve.fasta_2803
VFG043146(gi:28901391)	(fliF) flagellar MS-ring protein [lateral flagella (AI142)] [Vibrio parahaemolyticus RIMD 2210633]	c_amazonense.gapfilled_200_mauve.fasta_2440
VFG043152(gi:28901397)	(fliP) flagellar biosynthesis protein FliP [lateral flagella (AI142)] [Vibrio parahaemolyticus RIMD 2210633]	c_amazonense.gapfilled_200_mauve.fasta_2444
VFG043157(gi:28901403)	(lafA) lateral flagellin LafA [lateral flagella (AI142)] [Vibrio parahaemolyticus RIMD 2210633]	c_amazonense.gapfilled_200_mauve.fasta_2452
VFG043158(gi:28901405)	(lafB) flagellar distal capping protein LafB [lateral flagella (AI142)] [Vibrio parahaemolyticus RIMD 2210633]	c_amazonense.gapfilled_200_mauve.fasta_2435
VFG043159(gi:28901406)	(lafC) LafC protein [lateral flagella (AI142)] [Vibrio parahaemolyticus RIMD 2210633]	c_amazonense.gapfilled_200_mauve.fasta_2434
VFG043164(gi:28901411)	(motA) flagellar motor protein MotA [lateral flagella (AI142)] [Vibrio parahaemolyticus RIMD 2210633]	c_amazonense.gapfilled_200_mauve.fasta_2429
VFG043165(gi:28901412)	(lafU/motB) chemotaxis LafU protein [lateral flagella (AI142)] [Vibrio parahaemolyticus RIMD 2210633]	c_amazonense.gapfilled_200_mauve.fasta_2428
VFG043167(gi:74101639)	(fliN) FliNL [ND (AI143)] [Aeromonas hydrophila str. AH-3]	c_amazonense.gapfilled_200_mauve.fasta_2443
VFG043172(gi:74101644)	(flhA) FlhAL [ND (AI143)] [Aeromonas hydrophila str. AH-3]	c_amazonense.gapfilled_200_mauve.fasta_2448
VFG043183(gi:74101655)	(flgB) FlgBL [ND (AI143)] [Aeromonas hydrophila str. AH-3]	c_amazonense.gapfilled_200_mauve.fasta_2813
VFG043184(gi:74101656)	(flgC) FlgCL [ND (AI143)] [Aeromonas hydrophila str. AH-3]	c_amazonense.gapfilled_200_mauve.fasta_2812
VFG043186(gi:74101658)	(flgE) FlgEL [ND (AI143)] [Aeromonas hydrophila str. AH-3]	c_amazonense.gapfilled_200_mauve.fasta_2810
VFG043206(gb|YP_001006774)	(cheY) chemotaxis regulatory protein CheY [peritrichous flagella (AI145)] [Yersinia enterocolitica subsp. enterocolitica 8081]	c_amazonense.gapfilled_200_mauve.fasta_2999
VFG043207(gb|YP_001006775)	(cheB) chemotaxis-specific methylesterase CheB [peritrichous flagella (AI145)] [Yersinia enterocolitica subsp. enterocolitica 8081]	c_amazonense.gapfilled_200_mauve.fasta_1809
VFG043207(gb|YP_001006775)	(cheB) chemotaxis-specific methylesterase CheB [peritrichous flagella (AI145)] [Yersinia enterocolitica subsp. enterocolitica 8081]	c_amazonense.gapfilled_200_mauve.fasta_894
VFG043211(gb|YP_001006780)	(cheA) chemotaxis protein CheA [peritrichous flagella (AI145)] [Yersinia enterocolitica subsp. enterocolitica 8081]	c_amazonense.gapfilled_200_mauve.fasta_1813
VFG043211(gb|YP_001006780)	(cheA) chemotaxis protein CheA [peritrichous flagella (AI145)] [Yersinia enterocolitica subsp. enterocolitica 8081]	c_amazonense.gapfilled_200_mauve.fasta_899
VFG043212(gb|YP_001006781)	(motB) flagellar motor protein MotB [peritrichous flagella (AI145)] [Yersinia enterocolitica subsp. enterocolitica 8081]	c_amazonense.gapfilled_200_mauve.fasta_1558
VFG043214(gi:218927893)	(flhB) flagellar biosynthesis protein FlhB [lateral flagella (AI146)] [Yersinia pestis CO92]	c_amazonense.gapfilled_200_mauve.fasta_2447
VFG043223(gi:218927902)	(fliG) flagellar motor switch protein G [lateral flagella (AI146)] [Yersinia pestis CO92]	c_amazonense.gapfilled_200_mauve.fasta_2439
VFG043224(gi:218927903)	(fliH) flagellar assembly protein H [lateral flagella (AI146)] [Yersinia pestis CO92]	c_amazonense.gapfilled_200_mauve.fasta_2438
VFG043225(gi:218927904)	(fliI) flagellum-specific ATP synthase [lateral flagella (AI146)] [Yersinia pestis CO92]	c_amazonense.gapfilled_200_mauve.fasta_2437
VFG043229(gi:218927911)	(flgD) flagellar basal body rod modification protein [lateral flagella (AI146)] [Yersinia pestis CO92]	c_amazonense.gapfilled_200_mauve.fasta_2811
VFG043231(gi:218927914)	(flgG) flagellar basal body rod protein FlgG [lateral flagella (AI146)] [Yersinia pestis CO92]	c_amazonense.gapfilled_200_mauve.fasta_2808
VFG043233(gi:218927916)	(flgI) flagellar basal body P-ring protein [lateral flagella (AI146)] [Yersinia pestis CO92]	c_amazonense.gapfilled_200_mauve.fasta_2806
VFG043244(gi:218927931)	(fliA) flagellar biosynthesis sigma factor [lateral flagella (AI146)] [Yersinia pestis CO92]	c_amazonense.gapfilled_200_mauve.fasta_2430
VFG043297(gi:126697822)	(fliI) flagellum-specific ATP synthase [HexNAc (AI148)] [Clostridium difficile 630]	c_amazonense.gapfilled_200_mauve.fasta_549
VFG043345(gb|YP_095788)	(fleR/flrC) sigma 54-dependent response regulator [polar flagella (AI149)] [Legionella pneumophila subsp. pneumophila str. Philadelphia 1]	c_amazonense.gapfilled_200_mauve.fasta_767

VFG043388(gi:15645899)	(fliZ) hypothetical protein [Pse5Ac7Ac (AI150)] [Helicobacter pylori 26695]	c_amazonense.gapfilled_200_mauve.fasta_2855
VFG043390(gb|NP_208373)	(pdxJ) pyridoxine 5'-phosphate synthase [Pse5Ac7Ac (AI150)] [Helicobacter pylori 26695]	c_amazonense.gapfilled_200_mauve.fasta_2160
VFG043391(gb|NP_208374)	(pdxA) 4-hydroxythreonine-4-phosphate dehydrogenase [Pse5Ac7Ac (AI150)] [Helicobacter pylori 26695]	c_amazonense.gapfilled_200_mauve.fasta_3731
VFG043394(gb|YP_002343725)	(cheA) chemotaxis histidine kinase [Pse5Ac7Ac, Pse5Ac7Am, Pse8OAc, Pse5Am7AcGlnAc (AI151)] [Campylobacter jejuni subsp. jejuni NCTC 11168]	c_amazonense.gapfilled_200_mauve.fasta_3001
VFG043534(gi:17988970)	(ugpB) glycerol-3-phosphate-binding periplasmic protein precursor [SP41/UgpB (AI309)] [Brucella melitensis bv. 1 str. 16M]	c_amazonense.gapfilled_200_mauve.fasta_2368
VFG043534(gi:17988970)	(ugpB) glycerol-3-phosphate-binding periplasmic protein precursor [SP41/UgpB (AI309)] [Brucella melitensis bv. 1 str. 16M]	c_amazonense.gapfilled_200_mauve.fasta_3240
VFG043537(gi:218562247)	(Cj0596) major antigenic peptide PEB-cell binding factor [Peb4/CBF2 (AI315)] [Campylobacter jejuni subsp. jejuni NCTC 11168]	c_amazonense.gapfilled_200_mauve.fasta_1663
VFG043537(gi:218562247)	(Cj0596) major antigenic peptide PEB-cell binding factor [Peb4/CBF2 (AI315)] [Campylobacter jejuni subsp. jejuni NCTC 11168]	c_amazonense.gapfilled_200_mauve.fasta_2401
VFG043537(gi:218562247)	(Cj0596) major antigenic peptide PEB-cell binding factor [Peb4/CBF2 (AI315)] [Campylobacter jejuni subsp. jejuni NCTC 11168]	c_amazonense.gapfilled_200_mauve.fasta_2402
VFG043537(gi:218562247)	(Cj0596) major antigenic peptide PEB-cell binding factor [Peb4/CBF2 (AI315)] [Campylobacter jejuni subsp. jejuni NCTC 11168]	c_amazonense.gapfilled_200_mauve.fasta_2403
VFG043551(gi:15827894)	(ML1683) histone-like protein [histone-like protein (Hlp)/laminin-binding protein (LBP) (AI354)] [Mycobacterium leprae TN]	c_amazonense.gapfilled_200_mauve.fasta_1227
VFG043551(gi:15827894)	(ML1683) histone-like protein [histone-like protein (Hlp)/laminin-binding protein (LBP) (AI354)] [Mycobacterium leprae TN]	c_amazonense.gapfilled_200_mauve.fasta_1615
VFG043553(gi:294660356)	(MGA_1142) organic hydroperoxide resistance protein [MG1142 (AI361)] [Mycoplasma gallisepticum str. R(low)]	c_amazonense.gapfilled_200_mauve.fasta_123
VFG043573(gi:15605121)	(CT396) molecular chaperone DnaK [MOMP (AI392)] [Chlamydia trachomatis D/UW-3/CX]	c_amazonense.gapfilled_200_mauve.fasta_1017
VFG043573(gi:15605121)	(CT396) molecular chaperone DnaK [MOMP (AI392)] [Chlamydia trachomatis D/UW-3/CX]	c_amazonense.gapfilled_200_mauve.fasta_1441
VFG043573(gi:15605121)	(CT396) molecular chaperone DnaK [MOMP (AI392)] [Chlamydia trachomatis D/UW-3/CX]	c_amazonense.gapfilled_200_mauve.fasta_1630
VFG043648(gi:15598818)	(rpoS) RNA polymerase sigma factor RpoS [type IV pili (AI097)] [Pseudomonas aeruginosa PAO1]	c_amazonense.gapfilled_200_mauve.fasta_3714
VFG043679(gi:112292704)	(clbP) putative penicillin binding protein [colibactin (TX033)] [Escherichia coli O18:K1:H7 str. IHE3034]	c_amazonense.gapfilled_200_mauve.fasta_1199
VFG043683(gi:112292708)	(clbL) putative amidase [colibactin (TX033)] [Escherichia coli O18:K1:H7 str. IHE3034]	c_amazonense.gapfilled_200_mauve.fasta_3853
VFG043688(gi:112292713)	(clbG) putative malonyl-CoA transacylase [colibactin (TX033)] [Escherichia coli O18:K1:H7 str. IHE3034]	c_amazonense.gapfilled_200_mauve.fasta_2974
VFG043691(gi:112292716)	(clbD) putative 3-hydroxyacyl-CoA dehydrogenase [colibactin (TX033)] [Escherichia coli O18:K1:H7 str. IHE3034]	c_amazonense.gapfilled_200_mauve.fasta_2237
VFG043799(gi:53720009)	(BPSL2403) non-hemolytic phospholipase C precursor [Plc-1 (TX168)] [Burkholderia pseudomallei K96243]	c_amazonense.gapfilled_200_mauve.fasta_754
VFG043813(gi:156976100)	(VIBHAR_04871) lipolytic enzyme [VHH (TX187)] [Vibrio harveyi ATCC BAA-1116]	c_amazonense.gapfilled_200_mauve.fasta_240
VFG044024(gb|ACA60977)	(ACA60977) alpha-toxin [alpha-toxin (septicum) (VF0416)] [Clostridium septicum str. NH2]	c_amazonense.gapfilled_200_mauve.fasta_1911
VFG044041(gi:197313281)	(vah1) Vah1 [VAH1 (TX460)] [Listonella anguillarum str. M93Sm]	c_amazonense.gapfilled_200_mauve.fasta_1747
VFG044081(gb|NP_250946)	(pvcC) paerucumarin biosynthesis protein PvcC [pyoverdine (IA001)] [Pseudomonas aeruginosa PAO1]	c_amazonense.gapfilled_200_mauve.fasta_565
VFG044082(gb|NP_250947)	(pvcD) paerucumarin biosynthesis protein PvcD [pyoverdine (IA001)] [Pseudomonas aeruginosa PAO1]	c_amazonense.gapfilled_200_mauve.fasta_3881
VFG044082(gb|NP_250947)	(pvcD) paerucumarin biosynthesis protein PvcD [pyoverdine (IA001)] [Pseudomonas aeruginosa PAO1]	c_amazonense.gapfilled_200_mauve.fasta_3917
VFG044083(gb|NP_250948)	(ptxR) transcriptional regulator PtxR [pyoverdine (IA001)] [Pseudomonas aeruginosa PAO1]	c_amazonense.gapfilled_200_mauve.fasta_1043
VFG044083(gb|NP_250948)	(ptxR) transcriptional regulator PtxR [pyoverdine (IA001)] [Pseudomonas aeruginosa PAO1]	c_amazonense.gapfilled_200_mauve.fasta_105
VFG044083(gb|NP_250948)	(ptxR) transcriptional regulator PtxR [pyoverdine (IA001)] [Pseudomonas aeruginosa PAO1]	c_amazonense.gapfilled_200_mauve.fasta_1058
VFG044083(gb|NP_250948)	(ptxR) transcriptional regulator PtxR [pyoverdine (IA001)] [Pseudomonas aeruginosa PAO1]	c_amazonense.gapfilled_200_mauve.fasta_1115
VFG044083(gb|NP_250948)	(ptxR) transcriptional regulator PtxR [pyoverdine (IA001)] [Pseudomonas aeruginosa PAO1]	c_amazonense.gapfilled_200_mauve.fasta_1249
VFG044083(gb|NP_250948)	(ptxR) transcriptional regulator PtxR [pyoverdine (IA001)] [Pseudomonas aeruginosa PAO1]	c_amazonense.gapfilled_200_mauve.fasta_1371
VFG044083(gb|NP_250948)	(ptxR) transcriptional regulator PtxR [pyoverdine (IA001)] [Pseudomonas aeruginosa PAO1]	c_amazonense.gapfilled_200_mauve.fasta_1426
VFG044083(gb|NP_250948)	(ptxR) transcriptional regulator PtxR [pyoverdine (IA001)] [Pseudomonas aeruginosa PAO1]	c_amazonense.gapfilled_200_mauve.fasta_1543
VFG044083(gb|NP_250948)	(ptxR) transcriptional regulator PtxR [pyoverdine (IA001)] [Pseudomonas aeruginosa PAO1]	c_amazonense.gapfilled_200_mauve.fasta_1617
VFG044083(gb|NP_250948)	(ptxR) transcriptional regulator PtxR [pyoverdine (IA001)] [Pseudomonas aeruginosa PAO1]	c_amazonense.gapfilled_200_mauve.fasta_2081
VFG044083(gb|NP_250948)	(ptxR) transcriptional regulator PtxR [pyoverdine (IA001)] [Pseudomonas aeruginosa PAO1]	c_amazonense.gapfilled_200_mauve.fasta_2277
VFG044083(gb|NP_250948)	(ptxR) transcriptional regulator PtxR [pyoverdine (IA001)] [Pseudomonas aeruginosa PAO1]	c_amazonense.gapfilled_200_mauve.fasta_2407
VFG044083(gb|NP_250948)	(ptxR) transcriptional regulator PtxR [pyoverdine (IA001)] [Pseudomonas aeruginosa PAO1]	c_amazonense.gapfilled_200_mauve.fasta_2879
VFG044083(gb|NP_250948)	(ptxR) transcriptional regulator PtxR [pyoverdine (IA001)] [Pseudomonas aeruginosa PAO1]	c_amazonense.gapfilled_200_mauve.fasta_3034
VFG044083(gb|NP_250948)	(ptxR) transcriptional regulator PtxR [pyoverdine (IA001)] [Pseudomonas aeruginosa PAO1]	c_amazonense.gapfilled_200_mauve.fasta_3117
VFG044083(gb|NP_250948)	(ptxR) transcriptional regulator PtxR [pyoverdine (IA001)] [Pseudomonas aeruginosa PAO1]	c_amazonense.gapfilled_200_mauve.fasta_3585
VFG044083(gb|NP_250948)	(ptxR) transcriptional regulator PtxR [pyoverdine (IA001)] [Pseudomonas aeruginosa PAO1]	c_amazonense.gapfilled_200_mauve.fasta_394
VFG044083(gb|NP_250948)	(ptxR) transcriptional regulator PtxR [pyoverdine (IA001)] [Pseudomonas aeruginosa PAO1]	c_amazonense.gapfilled_200_mauve.fasta_468
VFG044083(gb|NP_250948)	(ptxR) transcriptional regulator PtxR [pyoverdine (IA001)] [Pseudomonas aeruginosa PAO1]	c_amazonense.gapfilled_200_mauve.fasta_591
VFG044083(gb|NP_250948)	(ptxR) transcriptional regulator PtxR [pyoverdine (IA001)] [Pseudomonas aeruginosa PAO1]	c_amazonense.gapfilled_200_mauve.fasta_592
VFG044083(gb|NP_250948)	(ptxR) transcriptional regulator PtxR [pyoverdine (IA001)] [Pseudomonas aeruginosa PAO1]	c_amazonense.gapfilled_200_mauve.fasta_863
VFG044084(gi:15597579)	(PA2383) transcriptional regulator [pyoverdine (IA001)] [Pseudomonas aeruginosa PAO1]	c_amazonense.gapfilled_200_mauve.fasta_1670
VFG044084(gi:15597579)	(PA2383) transcriptional regulator [pyoverdine (IA001)] [Pseudomonas aeruginosa PAO1]	c_amazonense.gapfilled_200_mauve.fasta_1792
VFG044084(gi:15597579)	(PA2383) transcriptional regulator [pyoverdine (IA001)] [Pseudomonas aeruginosa PAO1]	c_amazonense.gapfilled_200_mauve.fasta_38
VFG044103(gi:15599903)	(phuS) hemin degrading factor [direct heme uptake system (IA049)] [Pseudomonas aeruginosa PAO1]	c_amazonense.gapfilled_200_mauve.fasta_3456
VFG044104(gi:15599904)	(phuR) heme/hemoglobin uptake outer membrane receptor PhuR precursor [direct heme uptake system (IA049)] [Pseudomonas aeruginosa PAO1]	c_amazonense.gapfilled_200_mauve.fasta_3455
VFG044110(gi:33591572)	(bhuV) hemin importer ATP-binding subunit [direct heme uptake system (IA046)] [Bordetella pertussis Tohama I]	c_amazonense.gapfilled_200_mauve.fasta_2736
VFG044121(gi:33593443)	(alcE) putative iron-sulfur protein [alcaligin (IA010)] [Bordetella pertussis Tohama I]	c_amazonense.gapfilled_200_mauve.fasta_1357
VFG044123(gi:33593445)	(alcS) putative drug resistance translocase [alcaligin (IA010)] [Bordetella pertussis Tohama I]	c_amazonense.gapfilled_200_mauve.fasta_1364
VFG044123(gi:33593445)	(alcS) putative drug resistance translocase [alcaligin (IA010)] [Bordetella pertussis Tohama I]	c_amazonense.gapfilled_200_mauve.fasta_3806
VFG044123(gi:33593445)	(alcS) putative drug resistance translocase [alcaligin (IA010)] [Bordetella pertussis Tohama I]	c_amazonense.gapfilled_200_mauve.fasta_623
VFG044136(gi:218927487)	(hmuU) hemin transport system permease HmuU [direct heme uptake system (IA058)] [Yersinia pestis CO92]	c_amazonense.gapfilled_200_mauve.fasta_3458
VFG044140(gi:218927856)	(hasF) outer membrane channel protein [HasA-type hemophore-mediated heme uptake system (IA044)] [Yersinia pestis CO92]	c_amazonense.gapfilled_200_mauve.fasta_1304
VFG044140(gi:218927856)	(hasF) outer membrane channel protein [HasA-type hemophore-mediated heme uptake system (IA044)] [Yersinia pestis CO92]	c_amazonense.gapfilled_200_mauve.fasta_3492
VFG044173(gi:28901507)	(pvuE) iron-dicitrate transporter ATP-binding subunit [vibrioferrin (IA038)] [Vibrio parahaemolyticus RIMD 2210633]	c_amazonense.gapfilled_200_mauve.fasta_3459
VFG044185(gi:37676958)	(VVA1298) phospho-2-dehydro-3-deoxyheptonate aldolase [vulnibactin (IA023)] [Vibrio vulnificus YJ016]	c_amazonense.gapfilled_200_mauve.fasta_1309
VFG044185(gi:37676958)	(VVA1298) phospho-2-dehydro-3-deoxyheptonate aldolase [vulnibactin (IA023)] [Vibrio vulnificus YJ016]	c_amazonense.gapfilled_200_mauve.fasta_66
VFG044190(gi:49474051)	(hutC) hemin ABC transporter, permease protein [direct heme uptake system (IA054)] [Bartonella quintana str. Toulouse]	c_amazonense.gapfilled_200_mauve.fasta_2735
VFG044201(gi:66045819)	(ascF) diaminobutyrate--2-oxoglutarate aminotransferase [achromobactin (IA003)] [Pseudomonas syringae pv. syringae B728a]	c_amazonense.gapfilled_200_mauve.fasta_3493
VFG044213(gi:47530081)	(isdF) iron compound ABC transporter ATP-binding protein [NEAT-type hemophore-mediated heme uptake system (IA039)] [Bacillus anthracis str. Ames Ancestor]	c_amazonense.gapfilled_200_mauve.fasta_824
VFG044238(gi:123440715)	(hemT) hemin-binding periplasmic protein [direct heme uptake system (IA059)] [Yersinia enterocolitica subsp. enterocolitica 8081]	c_amazonense.gapfilled_200_mauve.fasta_3457
VFG044242(gi:145300215)	(hutA) outer-membrane heme receptor [direct heme uptake system (IA062)] [Aeromonas salmonicida subsp. salmonicida A449]	c_amazonense.gapfilled_200_mauve.fasta_740
VFG044244(gi:145300217)	(ASA_3330) isochorismatase family protein [direct heme uptake system (IA062)] [Aeromonas salmonicida subsp. salmonicida A449]	c_amazonense.gapfilled_200_mauve.fasta_3738
VFG044254(gi:151220272)	(sbnA) O-Acetyl serine sulfhydrylase [staphyloferrin B (IA027)] [Staphylococcus aureus subsp. aureus str. Newman]	c_amazonense.gapfilled_200_mauve.fasta_2466
VFG044254(gi:151220272)	(sbnA) O-Acetyl serine sulfhydrylase [staphyloferrin B (IA027)] [Staphylococcus aureus subsp. aureus str. Newman]	c_amazonense.gapfilled_200_mauve.fasta_3088
VFG044280(gi:197284138)	(PMI0229) ABC transporter permease [Proteobactin (IA034)] [Proteus mirabilis HI4320]	c_amazonense.gapfilled_200_mauve.fasta_1316
VFG044281(gi:197284139)	(PMI0230) ABC transporter ATP-binding protein [Proteobactin (IA034)] [Proteus mirabilis HI4320]	c_amazonense.gapfilled_200_mauve.fasta_1315
VFG044289(gi:197284147)	(pbtG) substrate-binding protein [Proteobactin (IA034)] [Proteus mirabilis HI4320]	c_amazonense.gapfilled_200_mauve.fasta_1317
VFG044370(gi:33521628)	(qbsN) QbsN [thioquinolobactin (IA009)] [Pseudomonas fluorescens ATCC 17400]	c_amazonense.gapfilled_200_mauve.fasta_876
VFG044371(gi:40204913)	(qbsM) QbsM [thioquinolobactin (IA009)] [Pseudomonas fluorescens ATCC 17400]	c_amazonense.gapfilled_200_mauve.fasta_3311
VFG044378(gi:28192388)	(qbsC) QbsC [thioquinolobactin (IA009)] [Pseudomonas fluorescens ATCC 17400]	c_amazonense.gapfilled_200_mauve.fasta_3761
VFG044382(gi:28192392)	(qbsG) QbsG [thioquinolobactin (IA009)] [Pseudomonas fluorescens ATCC 17400]	c_amazonense.gapfilled_200_mauve.fasta_2851
VFG044389(gi:6959513)	(pdtorfF) putative sulfurylase [Pyridine-2,6-dithiocarboxylic acid (PDTC) (IA030)] [Pseudomonas stutzeri KC]	c_amazonense.gapfilled_200_mauve.fasta_142
VFG044396(gi:6959520)	(pdtorfM) putative aminotransferase [Pyridine-2,6-dithiocarboxylic acid (PDTC) (IA030)] [Pseudomonas stutzeri KC]	c_amazonense.gapfilled_200_mauve.fasta_1268
VFG044396(gi:6959520)	(pdtorfM) putative aminotransferase [Pyridine-2,6-dithiocarboxylic acid (PDTC) (IA030)] [Pseudomonas stutzeri KC]	c_amazonense.gapfilled_200_mauve.fasta_1330
VFG044396(gi:6959520)	(pdtorfM) putative aminotransferase [Pyridine-2,6-dithiocarboxylic acid (PDTC) (IA030)] [Pseudomonas stutzeri KC]	c_amazonense.gapfilled_200_mauve.fasta_3717
VFG044398(gi:6959522)	(pdtorfO) putative acyl-CoA dehydrogenase [Pyridine-2,6-dithiocarboxylic acid (PDTC) (IA030)] [Pseudomonas stutzeri KC]	c_amazonense.gapfilled_200_mauve.fasta_2763
VFG044427(gi:114416732)	(vabA) 2,3-dihydroxybenzoate-2,3-dehydrogenase [Vanchrobactin (IA025)] [Listonella anguillarum RV22]	c_amazonense.gapfilled_200_mauve.fasta_1930
VFG044431(gi:114416736)	(vabS) putative siderophore exporter [Vanchrobactin (IA025)] [Listonella anguillarum RV22]	c_amazonense.gapfilled_200_mauve.fasta_1320
VFG045297(gi:52840986)	(hisF) imidazole glycerol phosphate synthase subunit HisF [LPS (VF0171)] [Legionella pneumophila subsp. pneumophila str. Philadelphia 1]	c_amazonense.gapfilled_200_mauve.fasta_505
VFG045340(gb|NP_539653)	(ricA) Rab2 interacting conserved protein A [RicA (VF0414)] [Brucella melitensis bv. 1 str. 16M]	c_amazonense.gapfilled_200_mauve.fasta_726
VFG045346(gb|NP_933683)	(IlpA) immunogenic lipoprotein A [IlpA (VF0513)] [Vibrio vulnificus YJ016]	c_amazonense.gapfilled_200_mauve.fasta_1676
VFG045467(gb|YP_107884)	(cdpA) cyclic di-GMP phosphodiesterase [CdpA (VF0432)] [Burkholderia pseudomallei K96243]	c_amazonense.gapfilled_200_mauve.fasta_2220
VFG045476(gi:52840277)	(lpg0021) Dot/Icm type IV secretion system effector [Dot/Icm (VF0156)] [Legionella pneumophila subsp. pneumophila str. Philadelphia 1]	c_amazonense.gapfilled_200_mauve.fasta_2616
VFG045607(gb|YP_096929)	(lpg2936) Dot/Icm type IV secretion system effector [Dot/Icm (VF0156)] [Legionella pneumophila subsp. pneumophila str. Philadelphia 1]	c_amazonense.gapfilled_200_mauve.fasta_245
VFG045670(gi:383327725)	(EFAU004_00405) LacI family transcriptional regulator [BopD (CVF615)] [Enterococcus faecium Aus0004]	c_amazonense.gapfilled_200_mauve.fasta_2487
VFG045671(gi:529233057)	(EFAU085_00344) LacI family sugar-binding transcriptional regulator [BopD (CVF615)] [Enterococcus faecium Aus0085]	c_amazonense.gapfilled_200_mauve.fasta_2101
VFG045688(gi:383329042)	(uppS) undecaprenyl diphosphate synthase [Capsule (CVF618)] [Enterococcus faecium Aus0004]	c_amazonense.gapfilled_200_mauve.fasta_1585
VFG045692(gi:289163963)	(htpB) molecular chaperone GroEL [Hsp60 (CVF347)] [Legionella longbeachae NSW150]	c_amazonense.gapfilled_200_mauve.fasta_1476
VFG045705(gi:289165397)	(mip) macrophage infectivity potentiator [Mip (CVF349)] [Legionella longbeachae NSW150]	c_amazonense.gapfilled_200_mauve.fasta_1993
VFG045705(gi:289165397)	(mip) macrophage infectivity potentiator [Mip (CVF349)] [Legionella longbeachae NSW150]	c_amazonense.gapfilled_200_mauve.fasta_3368
VFG045705(gi:289165397)	(mip) macrophage infectivity potentiator [Mip (CVF349)] [Legionella longbeachae NSW150]	c_amazonense.gapfilled_200_mauve.fasta_572
VFG045721(gi:289163734)	(feoB) ferrous iron transport protein B [Ferrous iron transport (CVF359)] [Legionella longbeachae NSW150]	c_amazonense.gapfilled_200_mauve.fasta_3079
VFG045730(gi:289165092)	(relA) GTP pyrophosphokinase [RelA (CVF365)] [Legionella longbeachae NSW150]	c_amazonense.gapfilled_200_mauve.fasta_3285
